# The first report of porcine parvovirus 7 (PPV7) in Colombia demonstrates the presence of variants associated with modifications at the level of the VP2-capsid protein

**DOI:** 10.1371/journal.pone.0258311

**Published:** 2021-12-16

**Authors:** Diana S. Vargas-Bermudez, Santiago Rendon-Marin, Julian Ruiz-Saenz, Dario Mogollón, Jairo Jaime

**Affiliations:** 1 Facultad de Medicina Veterinaria y de Zootecnia, Departamento de Salud Animal, Centro de Investigación en Inmunología e Infectología Veterinaria (CI3V), Universidad Nacional de Colombia, Sede Bogotá, Bogotá, CP, Colombia; 2 Facultad de Medicina Veterinaria y Zootecnia, Grupo de Investigación en Ciencias Animales—GRICA, Universidad Cooperativa de Colombia, Sede Bucaramanga, Bucaramanga, Colombia; Defense Threat Reduction Agency, UNITED STATES

## Abstract

There are a wide variety of porcine parvoviruses (PPVs) referred to as PPV1 to PPV7. The latter was discovered in 2016 and later reported in some countries in America, Asia, and Europe. PPV7 as a pathogenic agent or coinfection with other pathogens causing disease has not yet been determined. In the present study, we report the identification of PPV7 for the first time in Colombia, where it was found retrospectively since 2015 in 40% of the provinces that make up the country (13/32), and the virus was ratified for 2018 in 4/5 provinces evaluated. Additionally, partial sequencing (nucleotides 380 to 4000) was performed of four Colombian strains completely covering the VP2 and NS1 viral genes. A sequence identity greater than 99% was found when comparing them with reference strains from the USA and China. In three of the four Colombian strains, an insertion of 15 nucleotides (five amino acids) was found in the PPV7-VP2 capsid protein (540–5554 nt; 180–184 aa). Based on this insertion, the VP2 phylogenetic analysis exhibited two well-differentiated evolutionarily related groups. To evaluate the impact of this insertion on the structure of the PPV7-VP2 capsid protein, the secondary structure of two different Colombian strains was predicted, and it was determined that the insertion is located in the coil region and not involved in significant changes in the structure of the protein. The 3D structure of the PPV7-VP2 capsid protein was determined by threading and homology modeling, and it was shown that the insertion did not imply a change in the shape of the protein. Additionally, it was determined that the insertion is not involved in suppressing a potential B cell epitope, although the increase in length of the epitope could affect the interaction with molecules that allow a specific immune response.

## Introduction

Parvoviruses affect both vertebrate and invertebrate hosts. They belong to the *Parvoviridae* family and are characterized by small nonenveloped viruses containing a single-stranded DNA (ssDNA) genome [1]. According to the latest taxonomic report of the International Committee on Taxonomy of Viruses—ICTV, the *Parvoviridae* family is divided into three subfamilies: *Parvovirinae*, *Densovirinae*, and *Hamaparvovirinae* [2]. Porcine parvoviruses (PPVs) are located in two subfamilies: *Parvovirinae* and *Hamaparvovirinae*. In the first subfamily are the genera *Bocaparvovirus* (porcine bocavirus), *Protoparvovirus* (PPV1), *Tetraparvovirus* (PPV2 and PPV3), and *Copiparvovirus* (PPV4, PPV5, and PPV6). In the second subfamily is the new genus *Chaphamarparvovirus* (PPV7) [2], which has also been reported in other species of vertebrates, such as rodents (mouse kidney parvovirus, murine chapparvovirus, rat parvovirus 2) [3,4], canines (cachavirus 1A and cachavirus 1B) [5], birds (turkey parvovirus 2, chicken chapparvovirus 2) [6,7], and bats (desmodus rotondus chapparvovirus) [8]. PPV7 was discovered in the USA in 2016 through metagenomic analysis of rectal swab samples from adult pigs. PPV7-ssDNA is 4103 nucleotides (nt) long and has two major open reading frames (ORFs): ORF1 codes for the nonstructural protein NS1, and ORF2 codes for the structural VP2-capsid protein [9]. Among these proteins, it is significant that the amino acid (aa) identity of NS1 between PPV7 and other PPVs is less than 30% [9].

PPV7 has been reported in a few regions of the world, and its prevalence is considered variable. The latter can be explained because, by not knowing the pathogenesis of PPV7 and not being clear about the affected organs and systems, tissues with low viral load may be being collected. In North America, serum samples, nasal swabs, lung lavage, and rectal swabs were reported in the USA, and a prevalence between 2–17.2% was established [9]. In Asia, China has a higher prevalence (20–32.8%) in three different provinces from serum and lung samples [10–12], while in Korea, a highly variable prevalence (1.2–74%) was found in samples of aborted pig fetuses, lung tissues from finishing pigs, and their semen [13,14]. In Europe, PPV7 has been reported in Poland (19–39%) from serum and stool samples [15] and in Sweden from tonsil samples [16]. For South America, PPV7 has only been reported in Brazil from metagenomic analyses performed in pig livers [17].

Another essential characteristic of parvoviruses is that they have evolved rapidly [18]. Studies have shown evolutionary rates (ER) of approximately 3–5 x 10^−4^ per site per year (pspy) in VP genes and 10^−5^ pspy in NS [19,20]. Particularly for PPV7, a higher ER has been found compared to other porcine parvoviruses [14,20,21]; for PPV7-NS1, an ER of 8.01 × 10^−4^ pspy was determined versus PPV1-NS1 of 3.03 × 10^−5^ pspy, while for PPV7-VP2, it was 2.19 × 10^−3^ pspy versus 10^−4^ pspy for PPV1-VP2 [20–22]. Similar to other parvoviruses, PPV7 has shown a higher ER in the VP2 gene than in the NS1 gene [21–23]. In the center of the PPV7-VP2 sequence, additions ranging from 3 to 15 nt have been found with the addition of aa [12], and the consequences of these additions have not yet been established and probably have implications given that the PPV7-VP2 capsid protein is the major antigenic component of the virus [24].

From a clinical perspective, PPVs are among the primary pathogens associated with sow reproductive failure [25]. Although a particular clinical symptom associated with PPV7 has not yet been determined, some studies have shown the presence of PPV7-ssDNA in aborted fetuses and semen; this has led to a possible association with reproductive failure [13,14]. Additionally, studies indicate that PPV7 may be an important pathogen in coinfection with other viruses, such as porcine circovirus 2 and 3 (PCV2 and PCV3). In one study, a synergistic effect was found between PCV2 and PPV7, where the PPV7 prevalence rate was higher in a PCV2-positive farm [10,12]. In another study, the level of PCV2-DNA by real-time PCR was significantly higher in serum positive for PPV7 [26]. Coinfection with PCV2/PPV7 can cause a presentation similar to coinfection with PCV2/PPV1; that is, it can induce and increase porcine circovirus-associated disease (PCVAD) [27,28]. Regarding PPV7/PCV3 coinfection, this was the most frequent in a study in China where different viral coinfections from lung samples were evaluated [11,29].

In this study, we present the first report of PPV7 in Colombia, where viruses were found in pig production farms, and their molecular and phyloevolutionary characteristics are shown. Likewise, the structural properties of the VP2-capsid protein affected by changes found in the PPV7-VP2 gene sequence of the isolates are analyzed and associated with their possible consequences.

## Results

### PPV7 has been present in Colombia since 2015, with a wide distribution in the provinces

In the present study, we used samples from a DNA sample bank collected in 2015 and 2018 and stored in the Animal Virology Laboratory of the National University of Colombia, Bogotá, Colombia, South America. In 2015, stool samples (n = 759) were collected from the 32 provinces that make up Colombia, and it was found that 40% (13/32) of the provinces were positive for PPV7-DNA and that 6.06% (46/759) of the samples were positive (Table 1, S1 Fig). In 2018, sera (n = 126) were collected from five provinces, finding PPV7-DNA in 80% (4/5) of the evaluated provinces and 21% of the sera were positive. Thus, of the 885 samples, PPV7-DNA was detected in 8.24%. By the production stage, for 2015 (stool samples), PPV7 was detected in weaned and fattener pigs, while in 2018 (sera samples), it was also detected in weaned and fattener pigs but also in sows.

**Table 1:** Detection of PPV7-DNA in commercial farms. By 2015 in the 32 provinces that make up Colombia from stool and by 2018 in five provinces from serum.

Province	PPV7-DNA positive/samples tested		Total
	2015	2018	
Amazonas	0/5	-	0/5
Antioquia	4/72	0/9	4/81 (4.9%)
Arauca	0/17	-	0/17
Atlántico	0/7	11/31	11/38 (28.9%)
Boyacá	0/33	-	0/33
Bolivar	7/35	-	7/35 (20%)
Caldas	1/17	-	1/17 (8.88%)
Caquetá	6/34	-	6/34 (17%)
Casanare	5/21	-	5/21 (23.8%)
Cauca	0/9	-	0/9
Cesar	0/26	-	0/26
Choco	0/2	-	0/2
Cordoba	8/81	-	8/81 (9.87%)
Cundinamarca	6/49	4/38	10/87 (11.49%)
Guainía	0/5	-	0/5
Guajira	0/20	-	0/20
Guaviare	0/10	-	0/10
Huila	0/26	-	0/26
Magdalena	1/22	-	1/22 (4.54%)
Meta	0/2	-	0/2
Nariño	4/62	-	4/62 (6.45%)
Norte de Santander	0/72	-	0/72
Putumayo	0/13	-	0/13
Quindío	0/2	-	0/2
Risaralda	1/3	6/28	7/31/(22.5%)
San Andres	1/5	-	1/5 (20%)
Santander	0/23	-	0/23
Sucre	1/32	-	1/32 (3.12%)
Tolima	1/34	-	1/34 (2.94%)
Valle	0/9	6/20	6/29 (20.6%)
Vaupes	0/6	-	0/6
Vichada	0/5	-	0/5
**Total**	**46/759 (6%)**	**27/126 (21.4%)**	**73/885 (8.24%)**

(-) Province not evaluated (no samples).

### Phylogenetic analysis shows that the isolates found in Colombia are located in two groups differentiated by the presence or absence of an insertion of 15 nt in the VP2 gene

**Fig 1:**
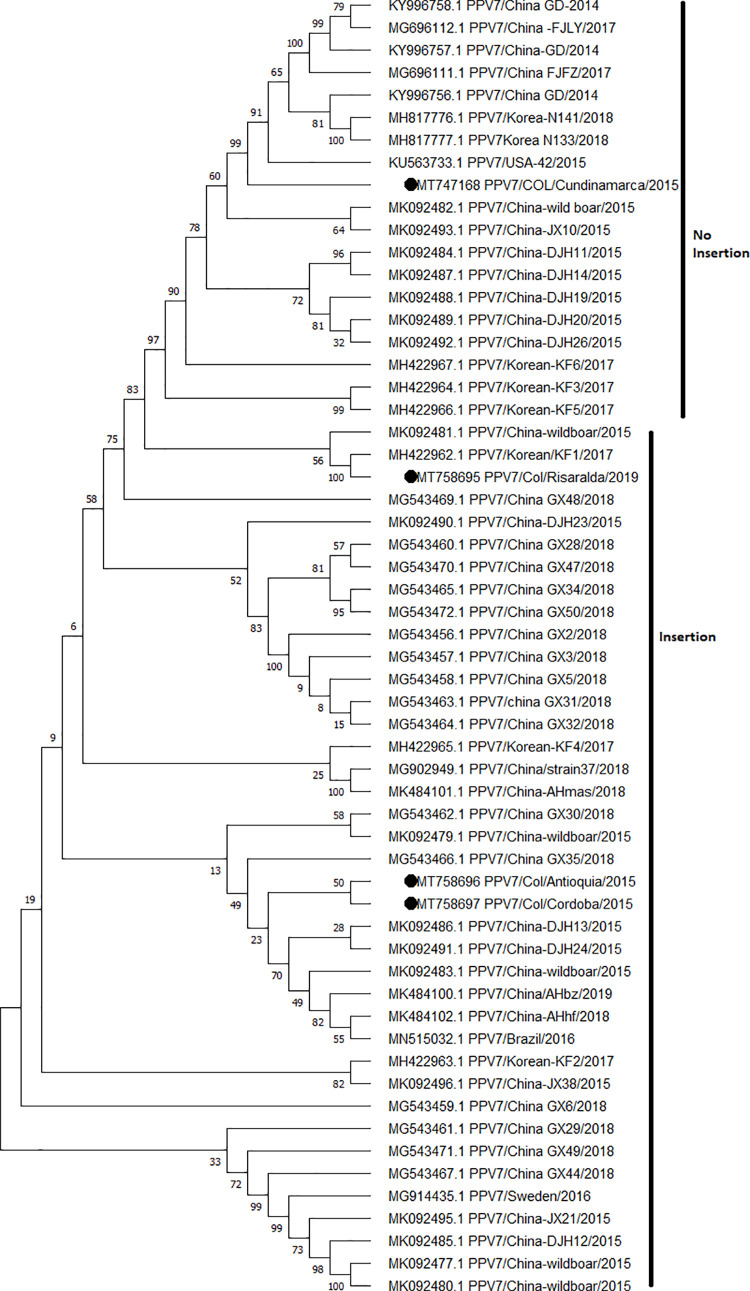
ML-method phylogenetic tree based on PPV7-VP2 nucleotide sequences. The phylogenetic tree with the highest log likelihood is exhibited. The analysis involved 58 nucleotide sequences and a total of 1369 positions in the final dataset. Groups are differentiated for the presence or absence of the five aa insertion.

The PPV7 genome was partially sequenced (nucleotides-nt 380 to 4000, which corresponds to 90% of the PPV7-DNA) from four samples: three from stool (PPV7/Col/Cundinamarca/2015, PPV7/Col/Antioquia/2015, and PPV7/Col/Cordoba/2015) accession number MT747168, MT758696, and MT758697, respectively; and a serum sample (PPV7/Col/Risaralda/2018) accession number MT758695. This sequencing completely covered the VP and NS genes. A phylogenetic tree analysis of partial PPV7-DNA genomes did not provide strong evidence for PPV7 genotyping (S2 Fig). Based on nt similarity analysis of the entire coding region, the four Colombian sequences shared 94.7–98% identity with each other and 99.73–99.8% nt sequence identity with reference strains from the USA (KU563733) and China (MG543458 and MG543466). A total of 58 PPV7-VP2 gene sequences with 1369 positions in the final dataset were retrieved from GenBank to carry out phylogenetic analysis through the maximum likelihood method (Fig 1 and S1 Table).

Comparative analysis of the PPV7-VP2 gene showed an identity of 91.9–98.2% for nt and 90.7–98% for aa between the Colombian strains. These sequences showed identities of 86–99.12% for nt and 83–99.53% for aa when compared to the USA (KU563733) and China (MG543458 and MG543466) strains. A striking result was that the VP2 gene of PPV7/Col/Cundinamarca/2015 showed a length of 1410 nt while the other three strains (PPV7/Col/Risaralda/2018, PPV7/Col/Antioquia/2015, and PPV7/Col/Cordoba/2015) were 1425 nt (S3 Fig) with an insertion of 15 nt (five aa) into VP2-capsid protein. This insertion is located between 540–554 nt (180–184 aa), translating to GEGAQ. The topology of the phylogenetic reconstruction exhibited two well-differentiated evolutionary-related groups. One group possessed an insertion composed of diverse stains from China, Korea, Sweden, Brazil, and the Colombian strains PPV7/Col/Risaralda/2018, PPV7/Col/Antioquia/2015, and PPV7/Col/Cordoba/2015. The other group without the insertion included strains from Korea, China, the USA, and PPV7/Col/Cundinamarca/2015. The results indicated that both strains with or without the insertion circulate in countries with reported PPV7 cases.

### The insertion of five aa in the VP2-capsid protein did not reveal significant changes in its structure by 2D and 3D modeling

**Fig 2:**
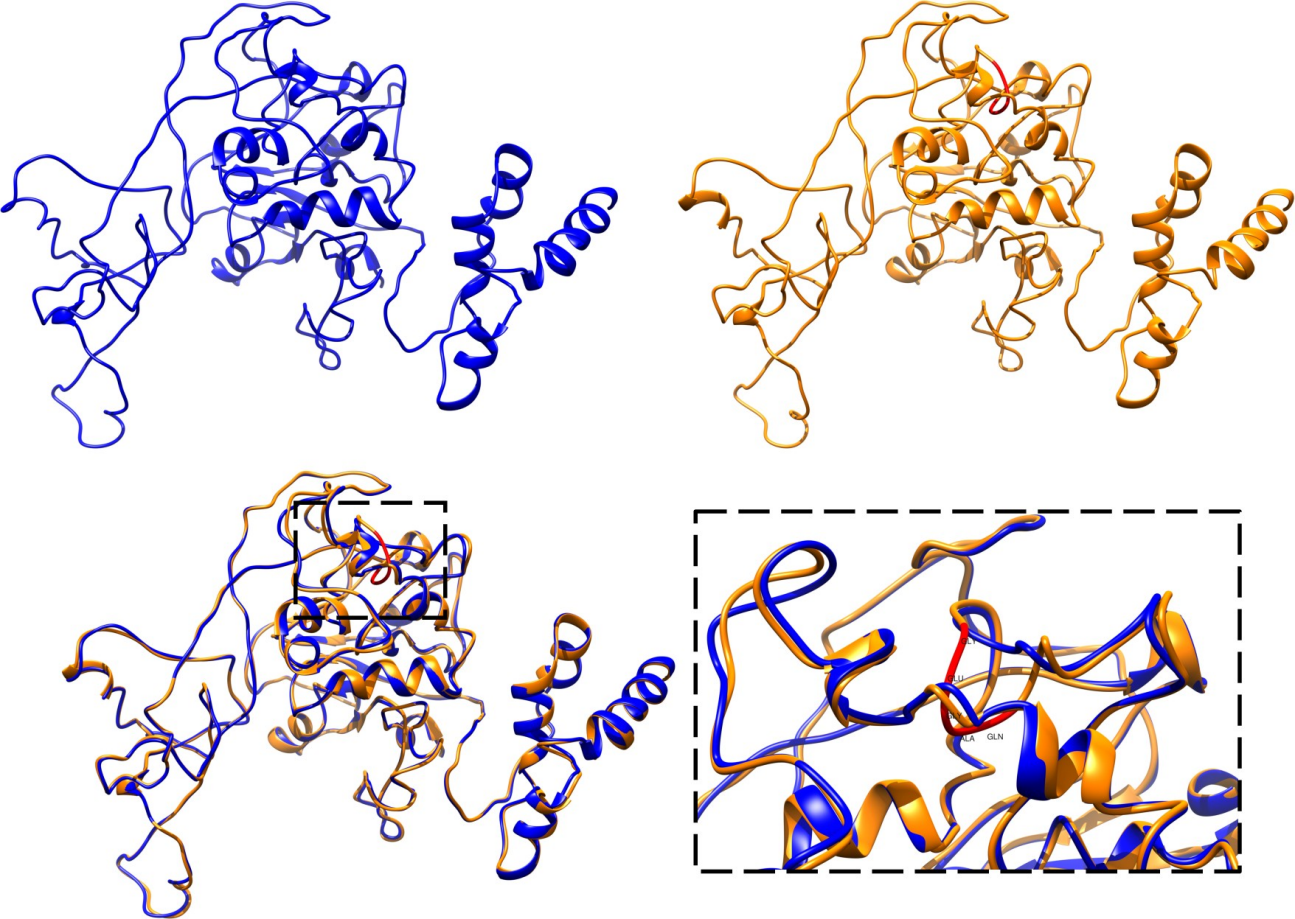
Structural analysis of PPV7/Col/Cundinamarca/2015 and PPV7/Col/Antioquia/2015 VP2-capsid protein models. **Upper left, t**hreading model for PPV7/Col/Cundinamarca/2015 VP2-capsid protein obtained by ITASSER. **Upper right, h**omology model for the PPV7/Col/Antioquia/2015 VP2-capsid protein elucidated with Modeler. In red is the five aa insertion in a coil region. **Bottom, s**tructural alignment of Colombian PPV7/Col/Cundinamarca/2015 (blue) and PPV7/Col/Antioquia/2015 (Orange) PPV7-VP2 capsid protein. Zoom: Five aa insertions colored red from the PPV7/Col/Antioquia/2015 VP2-capsid protein compared to the PPV7/Col/Cundinamarca/2015 VP2-capsid protein.

To evaluate the impact of the five aa insertion on the PPV7-VP2 capsid protein, the secondary structure of PPV7/Col/Antioquia/2015 (with insertion) and PPV7/Col/Cundinamarca/2015 (without insertion) was predicted. The overall secondary structure for PPV7-VP2 capsid protein strains was conserved despite the five aa insertion located through a coil region (S4 and S5 Figs). Therefore, this insertion does not confer a significant change to the protein secondary structure of Colombian PPV7. To determine structural changes in PPV7-VP2 capsid protein of Colombian strains with the insertion, the 3D structures of PPV7/Col/Antioquia/2015 and PPV7/Col/Cundinamarca/2015 VP2 capsid protein sequences were modeled and validated through computational tools. As shown in Table 2, both strain models have aa in an energetically favorable region for rotations and torsions with a percentage higher than 90% in the Ramachandran plot. Additionally, the overall model quality assayed by ProSA-Web indicated that both the PPV7/Col/Antioquia/2015 and PPV7/Col/Cundinamarca/2015 models were located within the range of scores likely found for proteins of similar size obtained by crystallographic approaches in the Protein Data Bank, calculated as the Z-value. Considering that the threading method employed by I-TASSER for elucidating the protein 3D structure yields the top 10 structures mainly employed for the model construction, the top structure was 1X9P, the crystallographic structure of the human adenovirus-2 capsid. The obtained models for PPV7/Col/Antioquia/2015 and PPV7/Col/Cundinamarca/2015 strains were carefully validated through computational tools, allowing further structural analysis (Table 2). The PPV7/Col/Antioquia/2015 and PPV7/Col/Cundinamarca/2015 VP2-capsid protein structures were elucidated through threading and homology modeling, respectively (Fig 2 upper left). As predicted in the secondary structure, the five aa insertion of PPV7/Col/Antioquia/2015 was located on a coil region (Fig 2 upper right). The overall structure for both PPV7/Col/Antioquia/2015 and PPV7/Col/Cundinamarca/2015 VP2-capsid protein exhibited a similar folding, represented in the structural alignment (Fig 2 bottom), and the TM-value was 0.996, indicating that the 3D structure of both proteins is identical. Both proteins carry out the same function in the viral particle and tropism. However, interaction capacity based on specific aa with cellular receptors could be differentiated between strains that lack the insertion. It is essential to mention that the GEGAQ (Gly, Glu, Gly, Ala, and Gln) insertion has a negative charge in Glu and can form hydrogen bonds with Gln, exhibiting potential interactions that could modify the binding of the protein with cellular receptors and neutralizing antibodies. Thus, elucidating the 3D structure of the PPV7-VP2 capsid protein helps determine the influence of structural changes, including five aa insertions and nonsynonymous aa changes, on the viral tropism of the humoral immune response.

**Table 2:** Computational validations of Colombian PPV7-VP2 capsid protein models.

Protein	Z Value^a^	Favorable region^b^ (%)	Verify 3D^c^ (%)
**PPV7/Col/Cundinamarca/2015**	-2,64	92,5	74,61
**PPV7/Col/Antioquia/2015**	-2,09	93,0	70,68

^a^Overall model quality based on 3D structures reported in the Protein Data Bank.

^b^Based on the Ramachandran plot that evaluates the rotations and torsion of amino acids.

^c^folding into a known three-dimensional structure.

### The insertion of five aa in the VP2-capsid protein can potentially affect the B cell epitope

The aa analysis of the PPV7-VP2 capsid protein in the Colombian sequences showed conserved loops present in other PPV7 strains; the Ca^2+^ binding loop was the 267YXGXXG272 motif and catalytic 300HDVPN304 motif of the putative secretory phospholipase A2 (PLA2), which have been reported in previous studies on PPV7 sequences [21]. To establish whether the five aa insertion in Colombian circulating PPV7 strains implies effects on the immune response to the PPV7-VP2 capsid protein, immunodominant epitopes for B cells were predicted using computational tools. A total of nine (9) and eight (8) B cell epitopes were predicted for PPV7/Col/Antioquia/2015 and PPV7/Col/Cundinamarca/2015 VP2-capsid protein, respectively. It is important to note that the five aa insertion of PPV7/Col/Antioquia/2015 VP2-capsid protein is located through a predicted epitope region (Table 3), similar to PPV7/Col/Cundinamarca/2015 VP2-capsid protein epitope prediction. The presence of the five aa insertion changed the length of the predicted epitope without disrupting the B cell epitope potential. Additionally, for the SVMTriP tool, there were different epitopes for each Colombian PPV7-VP2 capsid protein. We therefore concluded that the five aa insertion did not imply an impairment in the prediction of a potential B cell epitope; however, the sequence and length differences could mean changes in the antigenicity and immunodominance of this epitope.

**Table 3:** Predicted B cell epitopes from Colombian PPV7-VP2 capsid protein with and without insertion.

Protein	Length	Initial AA	Epitope	Tool
**PPV7/Col/Cundinamarca/2015 VP2-capsid protein**	12	18	DPYQYPTYKPFQ	BepiPred-2.0
	15	135	LVPKPTTATKEGVGNS	
	21	179	NKPYPTAGTTWPHTDAGTEQV	
	26	259	ESGYSHNIQNKKYQGPPGSRIVNENF	
	25	325	IEDKKVEKPQLGWPGTEWATPKYPP	
	19	419	TGGARRSWQARTRDTRDQQ	
	20	119	YEDTLWRSWYVAYKEGLVPK	SVMTriP
	20	224	NAMSFHWKTHGADEHCWYNL	
	20	402	MTHGIDSAFVLPTVRYRTGG	
**PPV7/Col/Antioquia/2015 VP2-capsid protein**	12	18	DPYQYPAYTPFQ	BepiPred-2.0
	16	135	LVPKRVPTKEGVGNSW	
	25	179	K**GEGAQ**AYPTAGTTWPHTDSGTEQV*	
	25	329	IEDKKVEKPQLGWPGTEWATPKYPP	
	19	423	TGGARRSWQARTRDARDQQ	
	20	36	YNTGWHILPNVLWRHFLSPK	SVMTriP
	20	228	NAMSFHWKTHGADEHCWYNL	
	20	406	MTHGIDSAFVLPTVRYRTGG	

*Predicted peptide including the five aa insertion.

## Discussion

PPV7 was identified for the first time in 2016 in the USA [9] and subsequently in other countries, such as China, South Korea, Sweden, Poland, and Brazil [10,13,15]. This study constitutes the first report of PPV7 in Colombia. Since its discovery, PPV7 has been found in different geographic regions and from a wide range of samples, such as serum, stool, aborted fetuses, semen, lung lavage, and nasal swabs. The above shows broad viral tropism, similar to that reported for PPV1 [30]. In retrospect, and supported by our results, PPV7 has been present in Colombia, at least since 2015, in 40% of the provinces that make up the country, indicating that the virus was already circulating in the country before it was officially reported—consistent with other retrospective studies in China, the USA, and Poland [9,10]. In our study, stool samples from 2015 were evaluated from all Colombian provinces, finding PPV7-DNA in 13/32 provinces. For 2018, the virus’s presence was confirmed by evaluating sera from five provinces, where four were positive. Comparing 32 provinces in 2015 vs. five provinces in 2018 does not offer us a panorama to determine if the prevalence has increased, but it does confirm the presence of the virus. Therefore, it is possible to have an effect on pig production systems in Colombia. In our study, the prevalence of PPV7-DNA in stool samples (6%) was lower than that reported in the USA and Poland (17% and 39%, respectively) [9,15]. In sera, it was higher (21%) than that reported for the same countries, but it was similar to that reported in China (27%) [12]. However, regarding the detection of PPV7 during the productive cycle, in one study, no PPV7 was found in suckling piglets, whereas it was detected in pigs older than five weeks of age, which could indicate the participation of passive immunity to control the virus in the first weeks of life of the piglets; similar result to that reported by Milek et al. 2018 [15]. This passive immunity behavior is similar to the response reported for PPV1, where the half-life of passively acquired antibodies has been determined to be between 19.7 and 29.0 days [31]. In the present study, the highest prevalence of PPV7 was found in the fattener group (70%), coinciding with the report of Milek et al. 2018 [15], while in sows, we found a prevalence of 22%. The high percentage of PPV7 detection in this last group could indicate a possible association of this virus with reproductive failure, as had been proposed by Ouh et al. 2018 [13], who reported PPV7 in 24% of aborted pig fetuses.

There are no previous studies that show a genotyping of PPV7. The only approximation is the study by Wang et al. 2020 [21], where 45 PPV7 sequences reported in GenBank (some complete and others partial) were analyzed, and two well-differentiated clades were determined. Additionally, they established a common ancestor for China’s strains dating back to 2004 by constructing an MCC tree-based. Likewise, the NS1 and VP2 regions were analyzed, but no similar clusters were found. In the present study, we carried out a phylogenetic analysis with ML trees for the PPV7-VP2 capsid protein from 58 strains reported worldwide, which showed two clades completely differentiated based on the presence or absence of the five aa insertion on this protein (Fig 1). It is important to note that this insert had not been considered in previous studies for the genotyping of PPV7 strains [21]. Of the four Colombian strains of PPV7 sequenced, in three, an insertion of five aa was found that coincided with strains reported in China, while in the fourth strain, the insertion was not found and presented greater identity with the reference strain from the USA (KU563733). These results could indicate a different origin between these strains and the convergence of these two origins in Colombia.

Once a pig is infected with parvovirus, the VP2-capsid structure plays an essential role in determining tropism [32] and is the primary antigenic determinant [33]. The sequences of the exposed surface loops are less conserved than the VP2 capsid internal regions, and changes in the aa sequence can influence receptor binding, pathogenicity, and antigenicity [22,34]. Lopez-Bueno et al. 2006 [35] reported that a single VP2-capsid aa change in parvovirus minute virus of mice (MVM) decreased receptor affinity and increased viral spread and disease severity. Therefore, it is of great importance to study the VP2-capsid protein of the newly reported parvoviruses and determine whether mutations at this level affect viral tropism and antigenicity. A previous study showed low identity (~ 11.6%) between PPV7-VP2 and the PPV1-VP2 capsid protein [21], which may indicate that PPV7 uses a different receptor to enter cells. The secondary structure of the PPV7-VP2 capsid protein has been determined for other strains elsewhere [21]. This is the first study in which the 3D structure of the PPV7-VP2 capsid protein was determined by threading and homology modeling. The above was done to determine structural changes that could imply an impairment in the process of viral adhesion to a cellular receptor and the immune response [22]. The insertion of five aa did not imply a change in the shape of the PPV7-VP2 capsid protein of the Colombian strains (Fig 2). However, the nature of the inserted aa sequence could mediate different interactions not only with cellular receptors but also with neutralizing antibodies that might influence the cellular tropism and adaptive immune response. Epitope search using computational tools enabled the determination of the five aa insertion on the Colombian PPV7-VP2 capsid protein and did not imply the suppression of a potential B cell epitope; however, the increase in length of the epitope could mean changes in the interaction capacity of this epitope with molecules that allow a specific immune response [36,37].

Similar to this study and other reports that found the insertion of five aa in the PPV7-VP2 capsid protein, other studies have reported a consecutive 9-nt deletion (1192–11200 bp) in the same gene, which leads to a three aa deletion (398–4400 aa) [10]. Both insertion and deletion could influence the immune response due to the emergence of new strains. In our study, nucleotide changes among strains circulating in Colombia imply differences in the aa sequence and, therefore, in the antigenic predictions with computational tools. Different studies evaluating the parvovirus VP2-capsid protein have shown that few substitutions can change viral tropism [38–42]. In conclusion, we reported strains with an insertion in the PPV7-VP2 capsid protein; although the 2D and 3D structure is maintained, this insertion can alter the receptor used, the interaction with immune cells, and pathogenesis. Therefore, we recommended carrying out *in vivo* studies where these changes are evaluated.

## Materials and methods

### Ethics statement

The Ethics Committee of Animal Care and Experimentation of Facultad de Medicina Veterinaria y de Zootecnia of the Universidad Nacional de Colombia, Sede Bogotá, approved this study (approval number CB-FMVZ-UN-011–20). The pigs sampled in this study were cared for following The Guiding Principle for Animal Care applied by the Asociación Colombiana de Porcicultores—PorkColombia.

### Sample collection

Colombia, South America, is administratively divided into 32 provinces. In 2015, 759 stool samples from commercial pig farms were collected from 32 provinces to determine, for that time, the presence of enteric viruses. The DNA extractions from these samples were stored at -70°C, constituting the sample bank that was used in the present study located at the Animal Virology Laboratory of the Facultad de Medicina Veterinaria y de Zootecnia, Universidad Nacional de Colombia, Sede Bogotá. In 2018 a new sampling was carried out. This time, 126 blood samples were randomly collected from 11 commercial pig farms located in five provinces corresponding to 1-, 2-, 3-, 7-, 11-, 15-, 19-, and 23-week-old pigs and sows. Likewise, the DNA extractions from these sera were frozen at -70°C and are part of the sample bank mentioned above. By the time the samples were collected, the stool samples were resuspended 1:5 in phosphate-buffered saline (PBS), vortexed for 5 min, and centrifuged at 10,000 x g for 10 min, while the blood samples were centrifuged at 5000 x g for 10 min, and the serum was recovered (five serum samples were pooled corresponding to one sample).

### Viral DNA extraction and PCR detection of PPV7

Viral DNA from 200 μl of pooled serum or stool samples was extracted using the High Pure Viral Nucleic Acid Kit (Roche®) according to the manufacturer’s instructions. Nucleic acids were eluted with 100 μl elution buffer and stored at -70°C (sample bank). For PPV7-DNA detection (year 2020), conventional PCR assays were performed using a set of PPV7 primers reported previously by Wang et al. 2019 [10] and targeting a 241 bp segment of the VP2 gene.

### Sequencing of PPV7 and sequence analysis

To characterize Colombian PPV7 strains, a set of overlapping primers was used (PPV7–380F and PPV7–1336R; PPV7–1270F and PPV7–2262R; PPV7 2158F and PPV7 3203R; PPV7–3022F and PPV7–4033R) as previously described by Wang et al. 2019 [10]. Reactions were performed in a total volume of 25 μl containing 0.25 μl of Accu Prime Taq (5 U/ml) (Thermo Fisher®), 1x AccuPrimer PCR Buffer (2.5 μl), 1 μl of each primer (20 μmol L − 1) and 2 μl of extracted DNA. The PCRs were performed on a BioRad®-DNA thermocycler using a protocol consisting of denaturation at 94°C for 2 min, followed by 35 cycles of denaturation at 94°C for 30 sec, annealing at 58°C for 30 sec and extension at 68°C for 1 min. PCR products were directly sequenced in both directions at the commercial sequencing facility SSiGMol (Sequencing and Molecular Analysis Service, Institute of Genetics, National University of Colombia, Bogotá). The chromatogram sequencing files were examined employing Chromas™ v2.6 (Technelysium, Helensvale, Australia), and four partial genomes (from 380 to 4000 nt) and consensus contigs of the PPV7-VP2 capsid protein from this study were assembled and edited using SeqMan Pro in Lasergene™ Software v.15 (Lasergene INC. Madison, Wisconsin, USA). Nucleotide BLAST (Basic Local Alignment Search Tool) was employed to search sequences available in the NCBI nucleotide databases. Four partial genome sequences were obtained with a partial genome (nt 380 to 4333), which included the PPV7-VP2 gene region. DNA sequence alignment was carried out with the ClustalW method and compared with other nucleotide sequences from PPV7 retrieved from GenBank. The best-fit model for nucleotide substitution was identified as a general time-reversible model with gamma-distributed rate heterogeneity and invariable sites (GTR + G + I). Phylogenetic reconstruction with a maximum-likelihood (ML) method was performed with a bootstrap value of 1000. The best model and phylogenetic analysis were performed by using MEGA ™ 7.0 for Windows® [43]. The Colombian PPV7 strains were designated PPV7/Col/Cundinamarca/2015, PPV7/Col/Risaralda/2018, PPV7/Col/Antioquia/2015, and PPV7/Col/Cordoba/2015 and deposited in GenBank under accession numbers MT747168, MT758695, MT758696, and MT758697, respectively.

### Structural analysis of PPV7-VP2 capsid proteins

The inferred aa sequences from the Colombian PPV7 strains were employed to determine the 3D structural differences. The secondary structure was predicted using PSIPRED [44]. The online tool I-TASSER [45–47] was employed to model the 3D structures of the PPV7/Col/Cundinamarca/2015 VP2-capsid protein, and the best-ranked structure in the hierarchical analysis was retrieved from the PDB. Then, PPV7/COL/Cundinamarca/2015 VP2-capsid protein was employed to elucidate PPV7/Col/Antioquia/2015 VP2-capsid protein through homology modeling with Modeler 9.24® [48]. The best models were refined through ModRefiner [49]. Refined structures were assessed and validated using bioinformatics tools. ProSA-Web was used to calculate the Z-score for the overall model quality compared to the PBD structures [50,51]. Ramachandran plots were constructed to determine aa in energetically favorable regions regarding dihedral angles ψ against φ of aa residues in novel protein structures [52]. The homology model for PPV7/Col/Antioquia/2015 VP2-capsid protein was structurally aligned to the template structure to establish the Root Mean Square Deviation (RMSD) differences utilizing TM-Align based on TM-score [53]. Finally, verify 3D was used to determine whether protein sequences fold into a known three-dimensional structure [54,55]. Molecular graphics were performed with UCSF Chimera [56].

### B cell epitope prediction of the PPV7-VP2 capsid protein

Linear B cell epitopes from the PPV7-VP2 capsid protein were predicted through computational tools such as BepiPred 2.0 from the Immune Epitope Database [57] with a threshold of 0.55 and higher than 10 aa in length and SVMTriP, a computational tool for predicting linear B cell antigenic epitopes with a 20 aa window search [58].

## Supporting information

S1 Fig Detection of PPV7 in Colombia. Map of Colombia, South America, where the 32 provinces are located. The provinces that were positive for PPV7 for 2015 (all provinces were evaluated) and for 2018 (five provinces were evaluated) are indicated. Two provinces (Cundinamarca and Risaralda) that were positive for PPV7 in both years were also identified. The map was downloaded from the National Department of Statistics of Colombia (DANE) database (http://geoportal.dane.gov.co/acerca-geoportal/acerca/), and the results were adapted to the map using the QGIS software available online (https://qgis.org/es/site/).
(PDF)

S2 Fig ML-method phylogenetic tree based on PPV7 partial genomes (from 380–400 nucleotides). The phylogenetic tree with the highest log likelihood is exhibited. The analysis involved 58 sequences. The Outgroup is a PPV1 nucleotide sequence.
(TIF)

S3 Fig Sequence alignment for the PPV7-VP2 gene from reference strains of four Colombian isolates. Comparative alignment of 54 reference sequences obtained in GenBank and the four Colombian isolates. The access numbers for each sequence and the registered name are included. For the Colombian strains MT758695, MT758696, and MT758696, the insertion of 15 nt in positions 540–5554 nt can be determined.
(PDF)

S4 Fig The secondary structure of the PPV7-VP2 capsid protein obtained from Colombian isolates. Secondary structure of the PPV7/Col/Cundinamarca/2015 VP2-capsid protein. Prediction was carried out with PSIPRED.
(TIF)

S5 Fig The secondary structure of the PPV7-VP2 capsid protein obtained from Colombian isolates. Secondary structure of the PPV7/Col/Antioquia 2015 VP2-capsid protein. Prediction was carried out with PSIPRED.
(TIF)

S1 Table Summary of the 54 reference PPV7 sequences compared with four Colombian sequences obtained in the present study. (DOCX)
