# Supplementary figures and images for: The first report of porcine parvovirus 7 (PPV7) in Colombia demonstrates the presence of variants associated with modifications at the level of the VP2-capsid protein
Source: PLoS One. 2021 Dec 16;16(12):e0258311. doi: 10.1371/journal.pone.0258311 (PMC8675767; doi:10.1371/journal.pone.0258311)

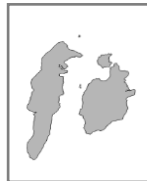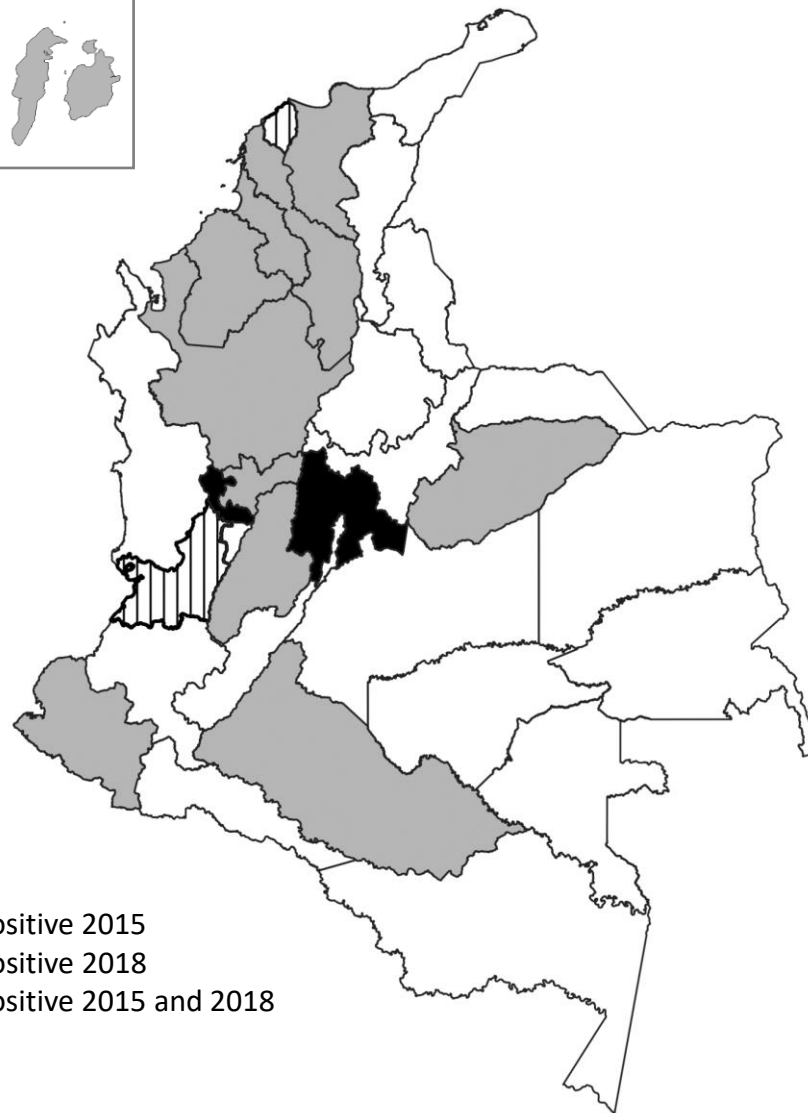

- PPV7 Positive 2015
- ▨ PPV7 Positive 2018
- PPV7 Positive 2015 and 2018

Supplement: S1 Fig — Map of Colombia, South America, where the 32 provinces are located. The provinces that were positive for PPV7 for 2015 (all provinces were evaluated) and for 2018 (five provinces were evaluated) are indicated. Two provinces (Cundinamarca and Risaralda) that were positive for PPV7 in both years were also identified. The map was downloaded from the National Department of Statistics of Colombia (DANE) database (http://geoportal.dane.gov.co/acerca-geoportal/acerca/), and the results were adapted to the map using the QGIS software available online (https://qgis.org/es/site/). (PDF) [file pone.0258311.s001.pdf]

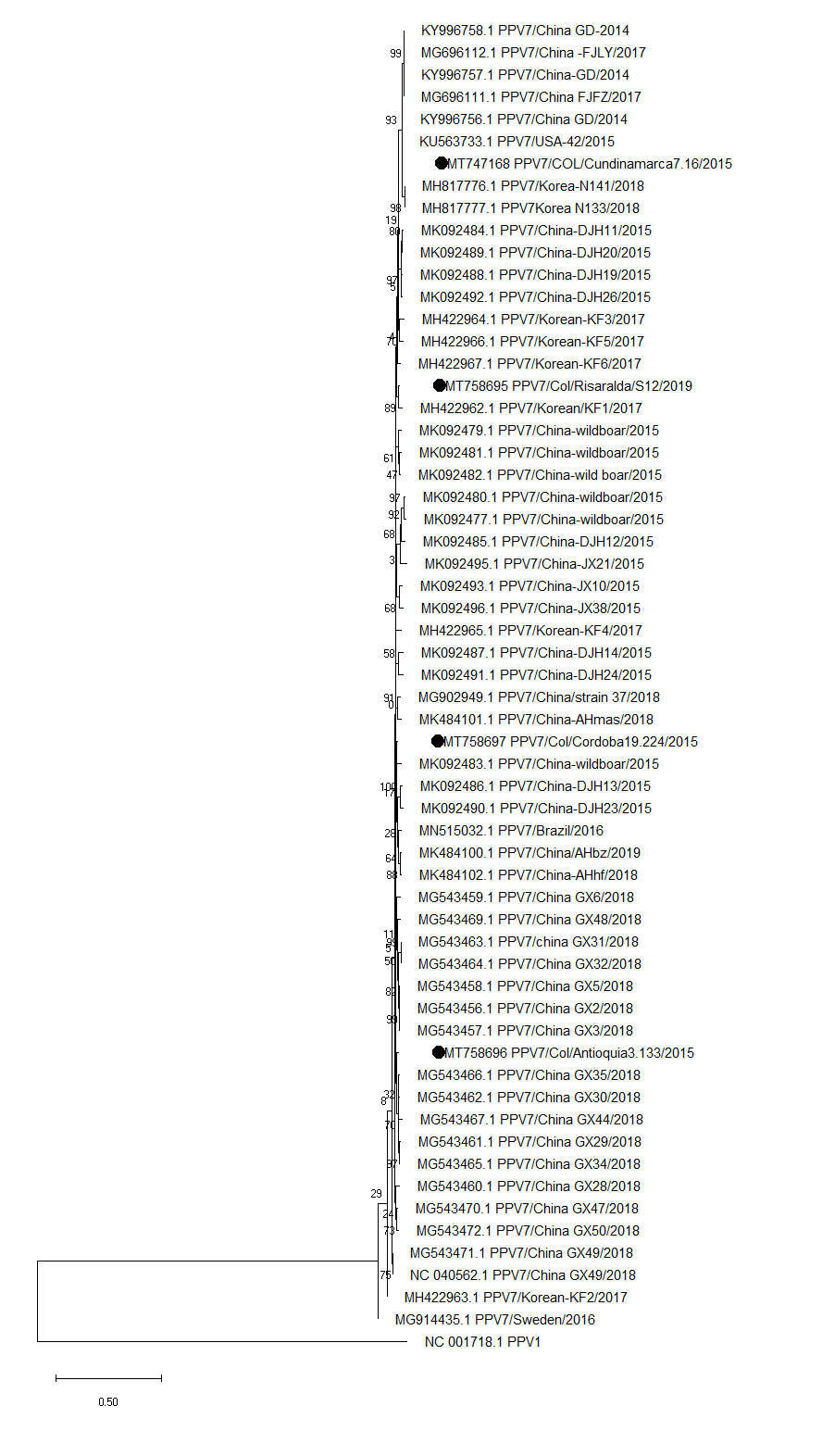

Supplement: S2 Fig — The phylogenetic tree with the highest log likelihood is exhibited. The analysis involved 58 sequences. The Outgroup is a PPV1 nucleotide sequence. (TIF) [file pone.0258311.s002.tif]

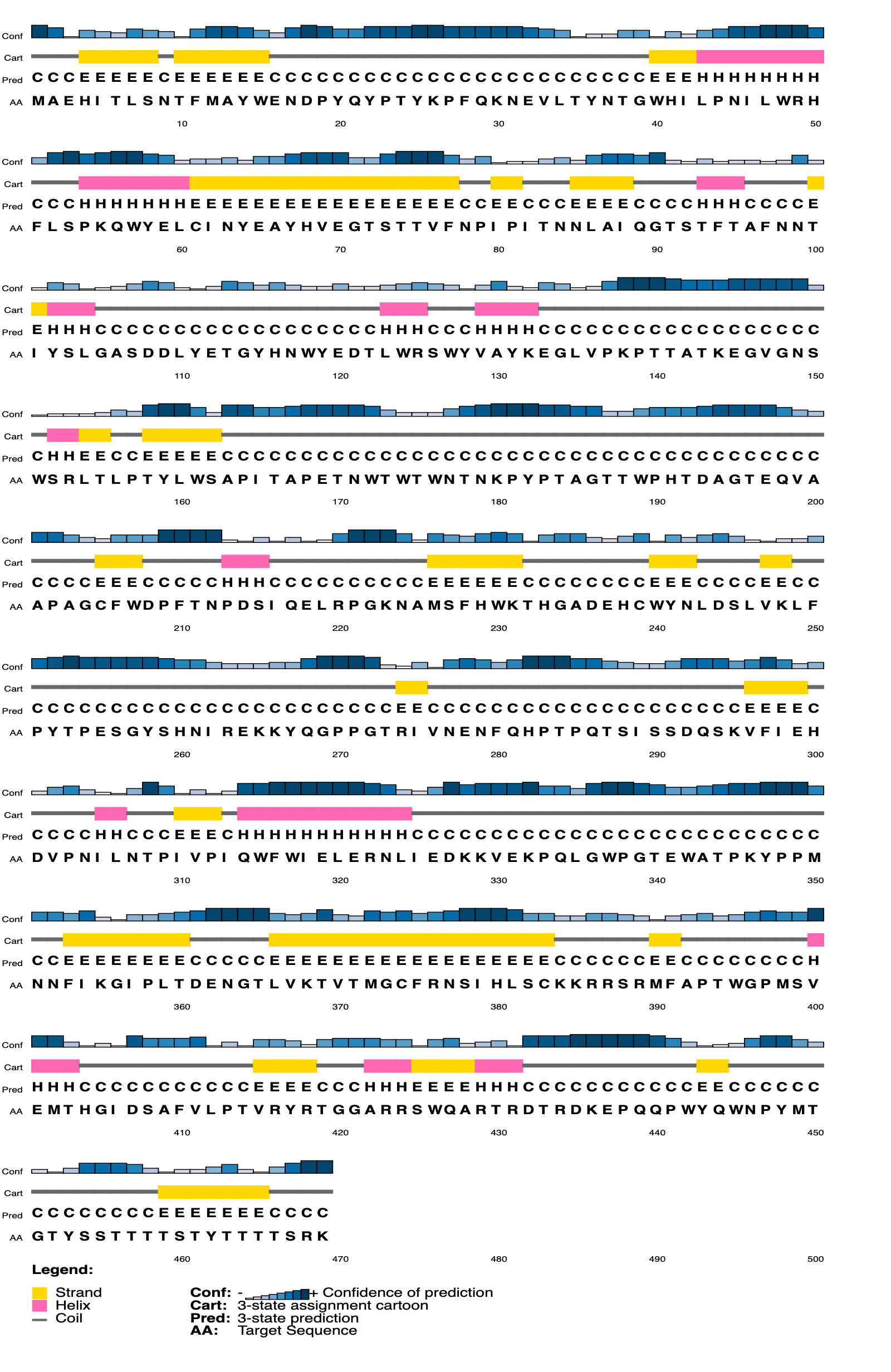

Supplement: S4 Fig — Secondary structure of the PPV7/Col/Cundinamarca/2015 VP2-capsid protein. Prediction was carried out with PSIPRED. (TIF) [file pone.0258311.s004.tif]

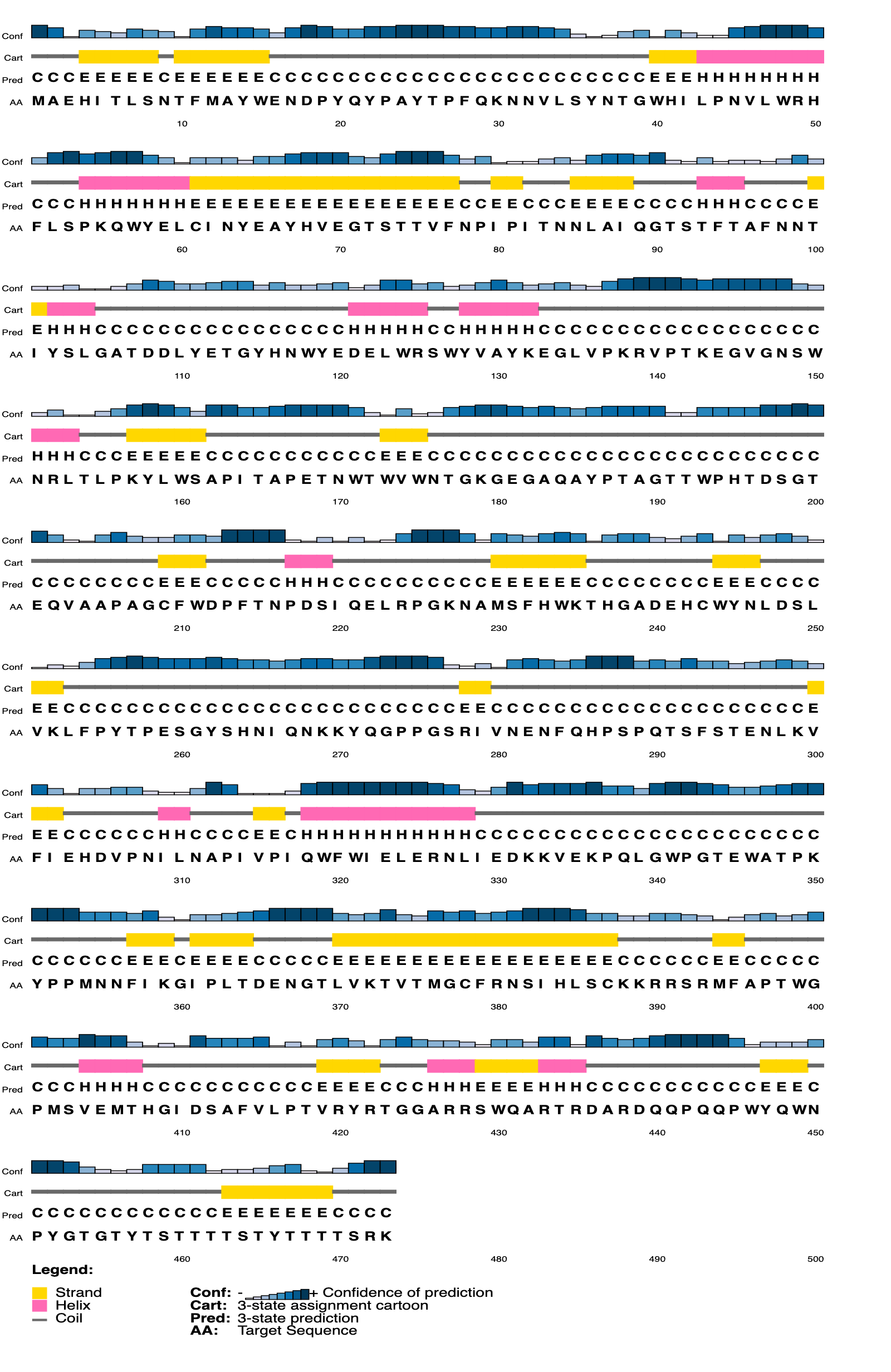

Supplement: S5 Fig — Secondary structure of the PPV7/Col/Antioquia 2015 VP2-capsid protein. Prediction was carried out with PSIPRED. (TIF) [file pone.0258311.s005.tif]
