# Supplementary material for: The first report of porcine parvovirus 7 (PPV7) in Colombia demonstrates the presence of variants associated with modifications at the level of the VP2-capsid protein
Source: PLoS One. 2021 Dec 16;16(12):e0258311. doi: 10.1371/journal.pone.0258311 (PMC8675767; doi:10.1371/journal.pone.0258311)
Supplement: S3 Fig — Comparative alignment of 54 reference sequences obtained in GenBank and the four Colombian isolates. The access numbers for each sequence and the registered name are included. For the Colombian strains MT758695, MT758696, and MT758696, the insertion of 15 nt in positions 540–5554 nt can be determined. (PDF) [file pone.0258311.s003.pdf]

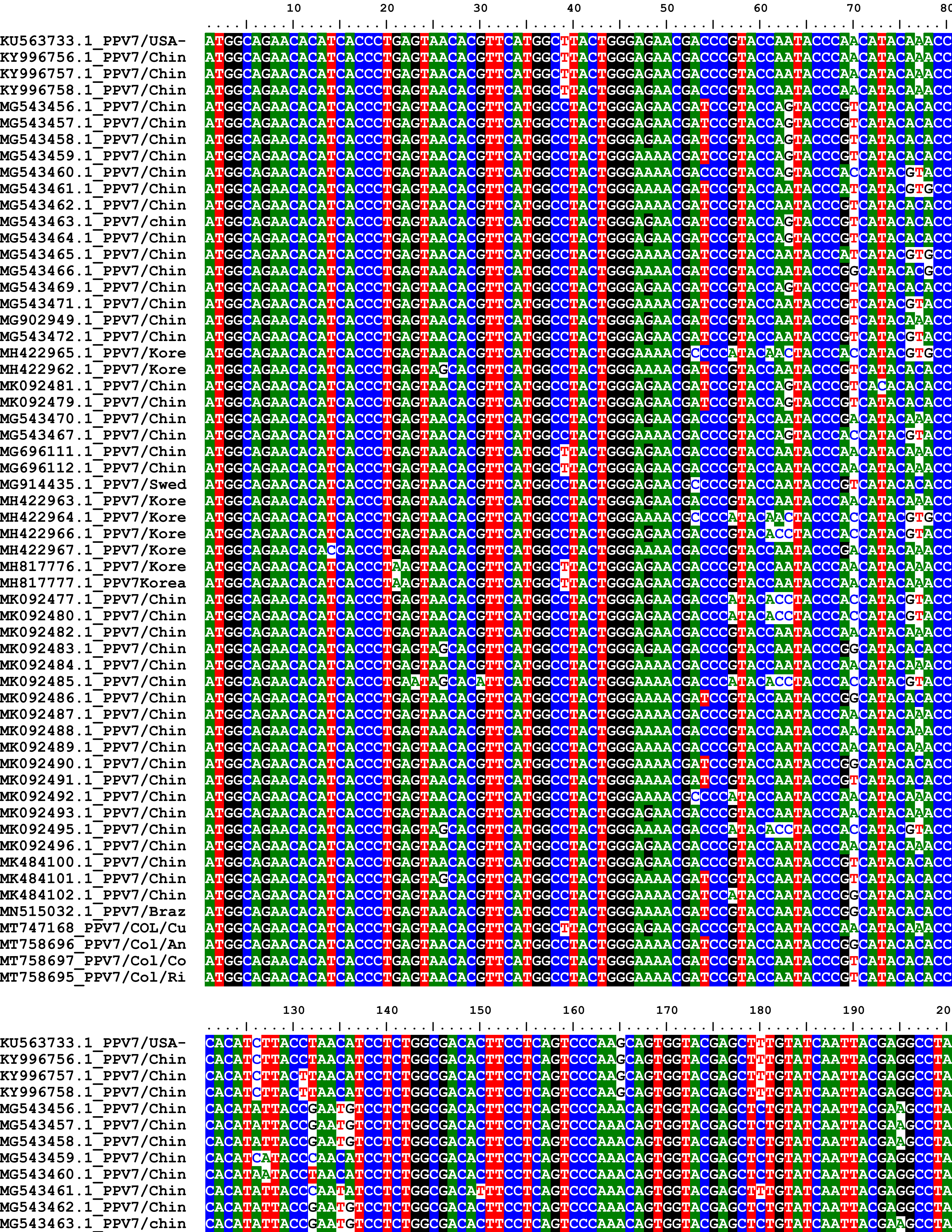
10 20 30 40 50 60 70 80
KU563733.1_PPV7/USA-
KY996756.1_PPV7/Chin
KY996757.1_PPV7/Chin
KY996758.1_PPV7/Chin
MG543456.1_PPV7/Chin
MG543457.1_PPV7/Chin
MG543458.1_PPV7/Chin
MG543459.1_PPV7/Chin
MG543460.1_PPV7/Chin
MG543461.1_PPV7/Chin
MG543462.1_PPV7/Chin
MG543463.1_PPV7/chin
MG543464.1_PPV7/Chin
MG543465.1_PPV7/Chin
MG543466.1_PPV7/Chin
MG543469.1_PPV7/Chin
MG543471.1_PPV7/Chin
MG902949.1_PPV7/Chin
MG543472.1_PPV7/Chin
MH422965.1_PPV7/Kore
MH422962.1_PPV7/Kore
MK092481.1_PPV7/Chin
MK092479.1_PPV7/Chin
MG543470.1_PPV7/Chin
MG543467.1_PPV7/Chin
MG696111.1_PPV7/Chin
MG696112.1_PPV7/Chin
MG914435.1_PPV7/Swed
MH422963.1_PPV7/Kore
MH422964.1_PPV7/Kore
MH422966.1_PPV7/Kore
MH422967.1_PPV7/Kore
MH817776.1_PPV7/Kore
MH817777.1_PPV7Korea
MK092477.1_PPV7/Chin
MK092480.1_PPV7/Chin
MK092482.1_PPV7/Chin
MK092483.1_PPV7/Chin
MK092484.1_PPV7/Chin
MK092485.1_PPV7/Chin
MK092486.1_PPV7/Chin
MK092487.1_PPV7/Chin
MK092488.1_PPV7/Chin
MK092489.1_PPV7/Chin
MK092490.1_PPV7/Chin
MK092491.1_PPV7/Chin
MK092492.1_PPV7/Chin
MK092493.1_PPV7/Chin
MK092495.1_PPV7/Chin
MK092496.1_PPV7/Chin
MK484100.1_PPV7/Chin
MK484101.1_PPV7/Chin
MK484102.1_PPV7/Chin
MN515032.1_PPV7/Braz
MT747168_PPV7/COL/Cu
MT758696_PPV7/Col/An
MT758697_PPV7/Col/Co
MT758695_PPV7/Col/Ri
130 140 150 160 170 180 190 20
KU563733.1_PPV7/USA-
KY996756.1_PPV7/Chin
KY996757.1_PPV7/Chin
KY996758.1_PPV7/Chin
MG543456.1_PPV7/Chin
MG543457.1_PPV7/Chin
MG543458.1_PPV7/Chin
MG543459.1_PPV7/Chin
MG543460.1_PPV7/Chin
MG543461.1_PPV7/Chin
MG543462.1_PPV7/Chin
MG543463.1_PPV7/chin

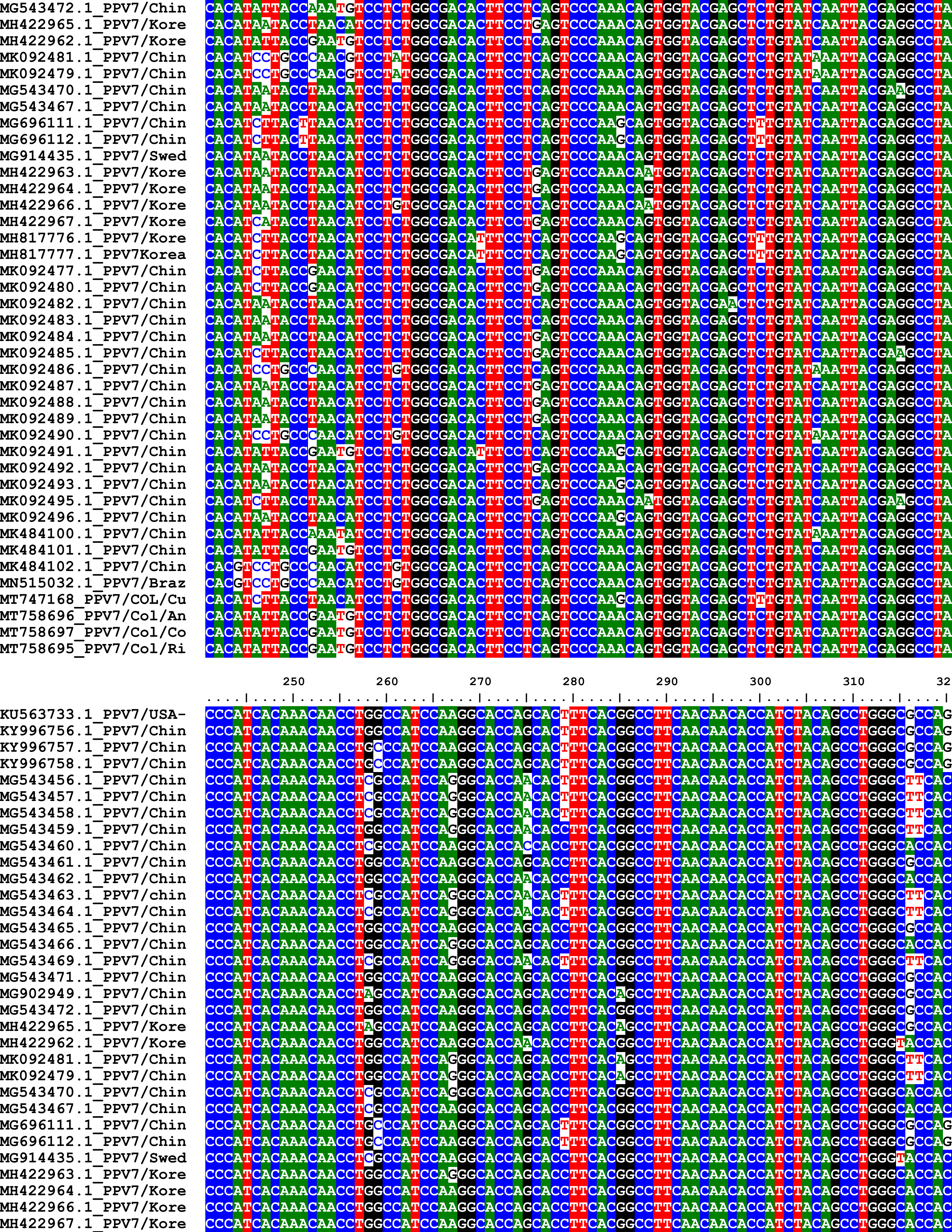
MG543472.1_PPV7/Chin
MH422965.1_PPV7/Kore
MH422962.1_PPV7/Kore
MK092481.1_PPV7/Chin
MK092479.1_PPV7/Chin
MG543470.1_PPV7/Chin
MG543467.1_PPV7/Chin
MG696111.1_PPV7/Chin
MG696112.1_PPV7/Chin
MG914435.1_PPV7/Swed
MH422963.1_PPV7/Kore
MH422964.1_PPV7/Kore
MH422966.1_PPV7/Kore
MH422967.1_PPV7/Kore
MH817776.1_PPV7/Kore
MH817777.1_PPV7Korea
MK092477.1_PPV7/Chin
MK092480.1_PPV7/Chin
MK092482.1_PPV7/Chin
MK092483.1_PPV7/Chin
MK092484.1_PPV7/Chin
MK092485.1_PPV7/Chin
MK092486.1_PPV7/Chin
MK092487.1_PPV7/Chin
MK092488.1_PPV7/Chin
MK092489.1_PPV7/Chin
MK092490.1_PPV7/Chin
MK092491.1_PPV7/Chin
MK092492.1_PPV7/Chin
MK092493.1_PPV7/Chin
MK092495.1_PPV7/Chin
MK092496.1_PPV7/Chin
MK484100.1_PPV7/Chin
MK484101.1_PPV7/Chin
MK484102.1_PPV7/Chin
MN515032.1_PPV7/Braz
MT747168_PPV7/COL/Cu
MT758696_PPV7/Col/An
MT758697_PPV7/Col/Co
MT758695_PPV7/Col/Ri
250
260
270
280
290
300
310
32
KU563733.1_PPV7/USA-
KY996756.1_PPV7/Chin
KY996757.1_PPV7/Chin
KY996758.1_PPV7/Chin
MG543456.1_PPV7/Chin
MG543457.1_PPV7/Chin
MG543458.1_PPV7/Chin
MG543459.1_PPV7/Chin
MG543460.1_PPV7/Chin
MG543461.1_PPV7/Chin
MG543462.1_PPV7/Chin
MG543463.1_PPV7/chin
MG543464.1_PPV7/Chin
MG543465.1_PPV7/Chin
MG543466.1_PPV7/Chin
MG543469.1_PPV7/Chin
MG543471.1_PPV7/Chin
MG902949.1_PPV7/Chin
MG543472.1_PPV7/Chin
MH422965.1_PPV7/Kore
MH422962.1_PPV7/Kore
MK092481.1_PPV7/Chin
MK092479.1_PPV7/Chin
MG543470.1_PPV7/Chin
MG543467.1_PPV7/Chin
MG696111.1_PPV7/Chin
MG696112.1_PPV7/Chin
MG914435.1_PPV7/Swed
MH422963.1_PPV7/Kore
MH422964.1_PPV7/Kore
MH422966.1_PPV7/Kore
MH422967.1_PPV7/Kore

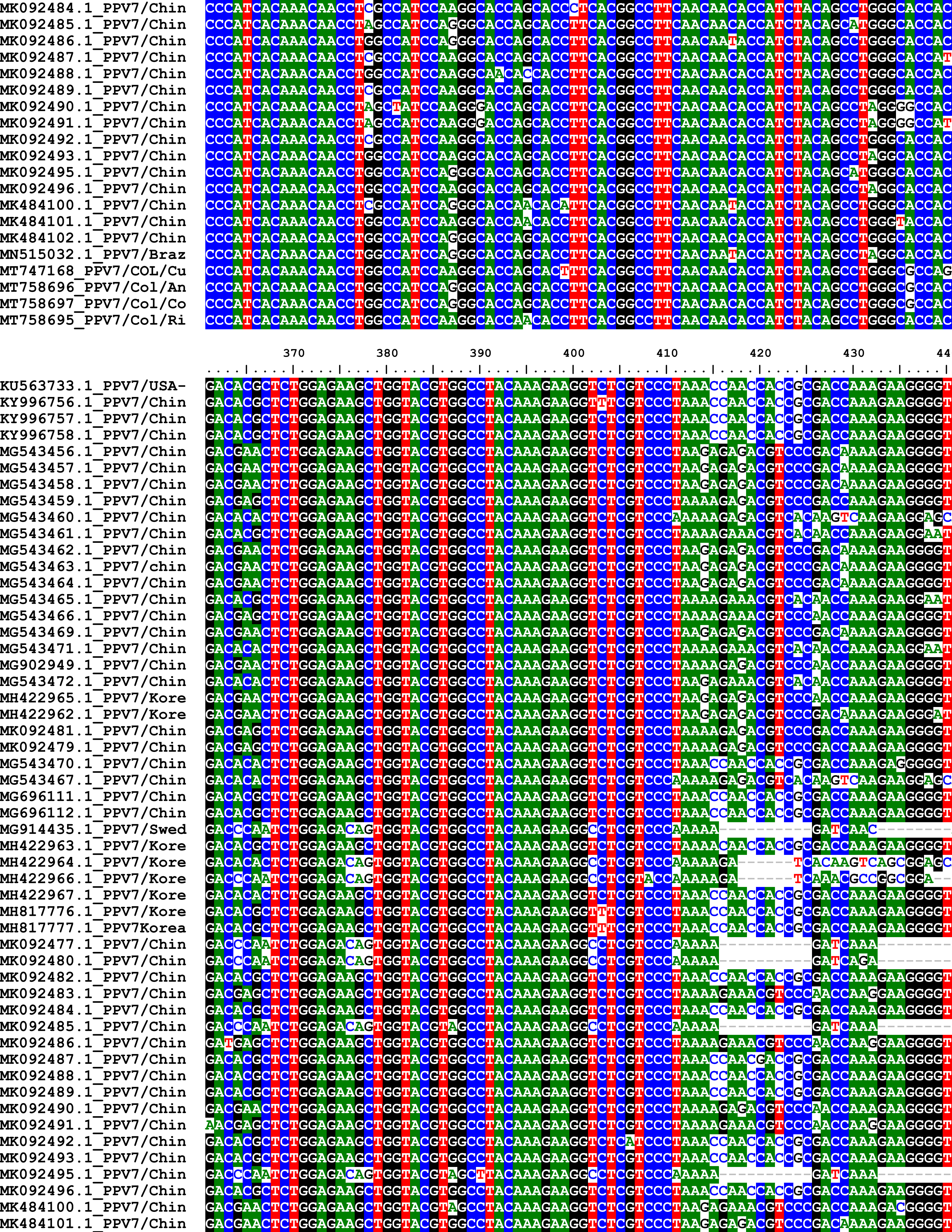
MK092484.1_PPV7/Chin
MK092485.1_PPV7/Chin
MK092486.1_PPV7/Chin
MK092487.1_PPV7/Chin
MK092488.1_PPV7/Chin
MK092489.1_PPV7/Chin
MK092490.1_PPV7/Chin
MK092491.1_PPV7/Chin
MK092492.1_PPV7/Chin
MK092493.1_PPV7/Chin
MK092495.1_PPV7/Chin
MK092496.1_PPV7/Chin
MK484100.1_PPV7/Chin
MK484101.1_PPV7/Chin
MK484102.1_PPV7/Chin
MN515032.1_PPV7/Braz
MT747168_PPV7/COL/Cu
MT758696_PPV7/Col/An
MT758697_PPV7/Col/Co
MT758695_PPV7/Col/Ri
370 380 390 400 410 420 430 44
KU563733.1_PPV7/USA-
KY996756.1_PPV7/Chin
KY996757.1_PPV7/Chin
KY996758.1_PPV7/Chin
MG543456.1_PPV7/Chin
MG543457.1_PPV7/Chin
MG543458.1_PPV7/Chin
MG543459.1_PPV7/Chin
MG543460.1_PPV7/Chin
MG543461.1_PPV7/Chin
MG543462.1_PPV7/Chin
MG543463.1_PPV7/chin
MG543464.1_PPV7/Chin
MG543465.1_PPV7/Chin
MG543466.1_PPV7/Chin
MG543469.1_PPV7/Chin
MG543471.1_PPV7/Chin
MG902949.1_PPV7/Chin
MG543472.1_PPV7/Chin
MH422965.1_PPV7/Kore
MH422962.1_PPV7/Kore
MK092481.1_PPV7/Chin
MK092479.1_PPV7/Chin
MG543470.1_PPV7/Chin
MG543467.1_PPV7/Chin
MG696111.1_PPV7/Chin
MG696112.1_PPV7/Chin
MG914435.1_PPV7/Swed
MH422963.1_PPV7/Kore
MH422964.1_PPV7/Kore
MH422966.1_PPV7/Kore
MH422967.1_PPV7/Kore
MH817776.1_PPV7/Kore
MH817777.1_PPV7Korea
MK092477.1_PPV7/Chin
MK092480.1_PPV7/Chin
MK092482.1_PPV7/Chin
MK092483.1_PPV7/Chin
MK092484.1_PPV7/Chin
MK092485.1_PPV7/Chin
MK092486.1_PPV7/Chin
MK092487.1_PPV7/Chin
MK092488.1_PPV7/Chin
MK092489.1_PPV7/Chin
MK092490.1_PPV7/Chin
MK092491.1_PPV7/Chin
MK092492.1_PPV7/Chin
MK092493.1_PPV7/Chin
MK092495.1_PPV7/Chin
MK092496.1_PPV7/Chin
MK484100.1_PPV7/Chin
MK484101.1_PPV7/Chin

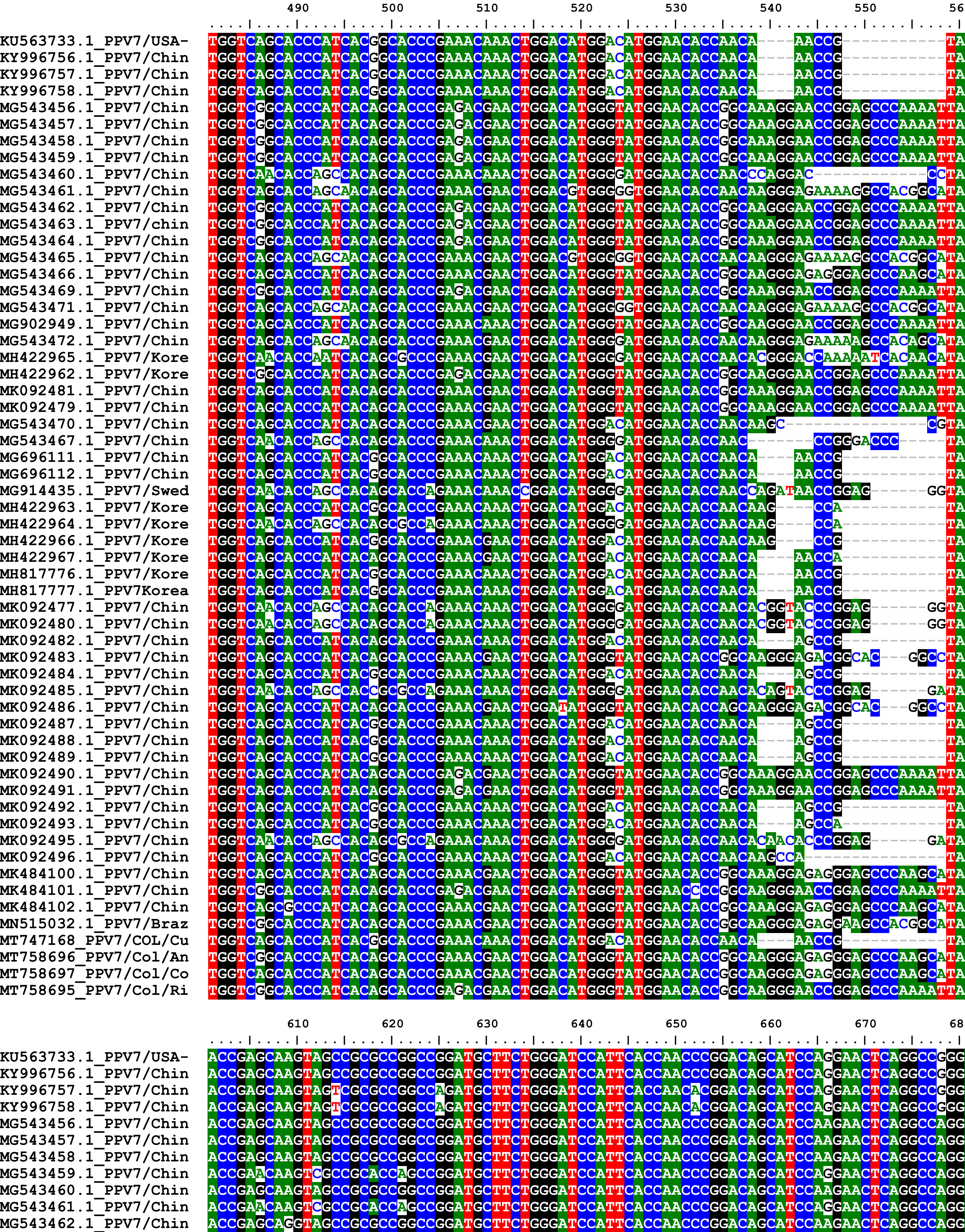
490 500 510 520 530 540 550 56
KU563733.1_PPV7/USA-
KY996756.1_PPV7/Chin
KY996757.1_PPV7/Chin
KY996758.1_PPV7/Chin
MG543456.1_PPV7/Chin
MG543457.1_PPV7/Chin
MG543458.1_PPV7/Chin
MG543459.1_PPV7/Chin
MG543460.1_PPV7/Chin
MG543461.1_PPV7/Chin
MG543462.1_PPV7/Chin
MG543463.1_PPV7/chin
MG543464.1_PPV7/Chin
MG543465.1_PPV7/Chin
MG543466.1_PPV7/Chin
MG543469.1_PPV7/Chin
MG543471.1_PPV7/Chin
MG902949.1_PPV7/Chin
MG543472.1_PPV7/Chin
MH422965.1_PPV7/Kore
MH422962.1_PPV7/Kore
MK092481.1_PPV7/Chin
MK092479.1_PPV7/Chin
MG543470.1_PPV7/Chin
MG543467.1_PPV7/Chin
MG696111.1_PPV7/Chin
MG696112.1_PPV7/Chin
MG914435.1_PPV7/Swed
MH422963.1_PPV7/Kore
MH422964.1_PPV7/Kore
MH422966.1_PPV7/Kore
MH422967.1_PPV7/Kore
MH817776.1_PPV7/Kore
MH817777.1_PPV7Korea
MK092477.1_PPV7/Chin
MK092480.1_PPV7/Chin
MK092482.1_PPV7/Chin
MK092483.1_PPV7/Chin
MK092484.1_PPV7/Chin
MK092485.1_PPV7/Chin
MK092486.1_PPV7/Chin
MK092487.1_PPV7/Chin
MK092488.1_PPV7/Chin
MK092489.1_PPV7/Chin
MK092490.1_PPV7/Chin
MK092491.1_PPV7/Chin
MK092492.1_PPV7/Chin
MK092493.1_PPV7/Chin
MK092495.1_PPV7/Chin
MK092496.1_PPV7/Chin
MK484100.1_PPV7/Chin
MK484101.1_PPV7/Chin
MK484102.1_PPV7/Chin
MN515032.1_PPV7/Braz
MT747168_PPV7/COL/Cu
MT758696_PPV7/Col/An
MT758697_PPV7/Col/Co
MT758695_PPV7/Col/Ri
610 620 630 640 650 660 670 68
KU563733.1_PPV7/USA-
KY996756.1_PPV7/Chin
KY996757.1_PPV7/Chin
KY996758.1_PPV7/Chin
MG543456.1_PPV7/Chin
MG543457.1_PPV7/Chin
MG543458.1_PPV7/Chin
MG543459.1_PPV7/Chin
MG543460.1_PPV7/Chin
MG543461.1_PPV7/Chin
MG543462.1_PPV7/Chin

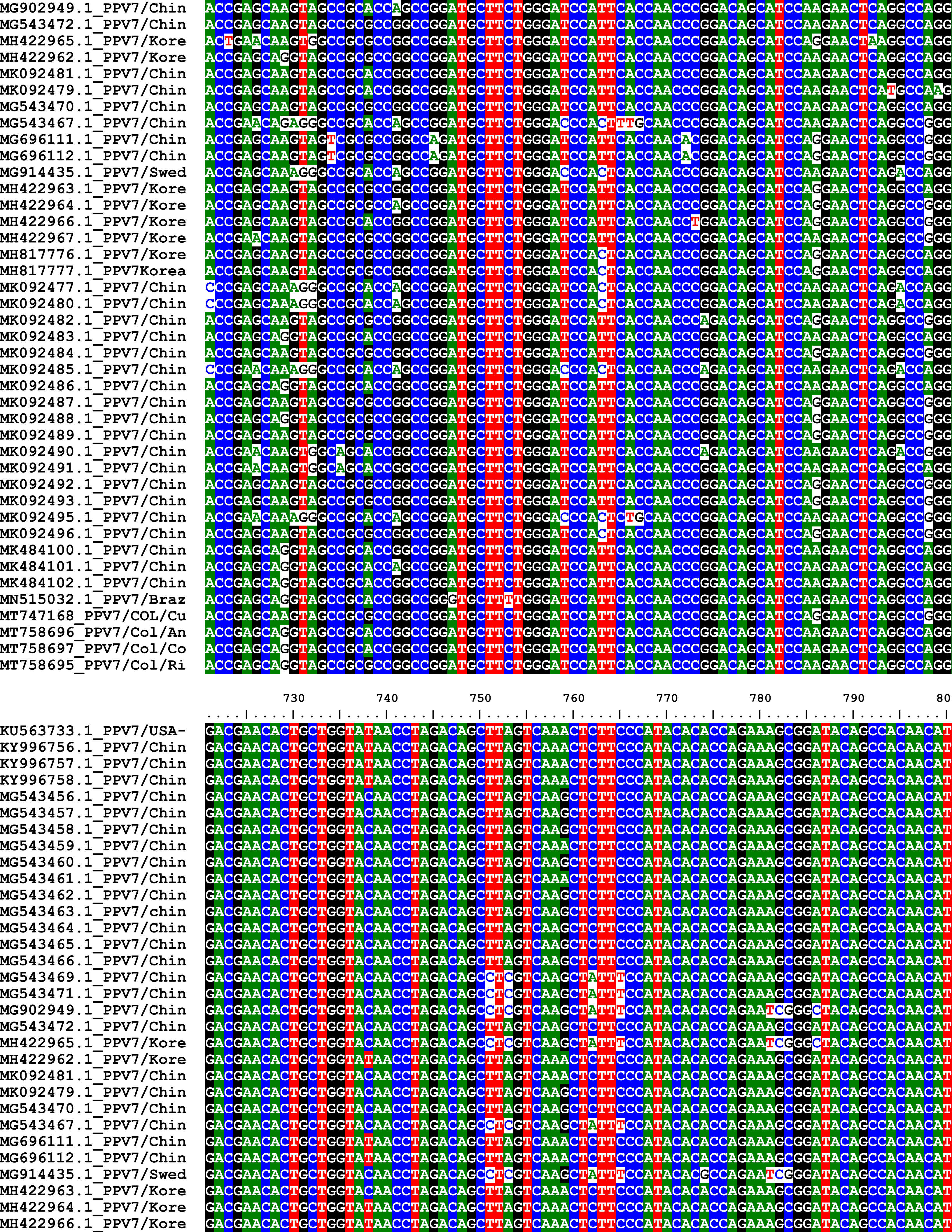
MG902949.1_PPV7/Chin
MG543472.1_PPV7/Chin
MH422965.1_PPV7/Kore
MH422962.1_PPV7/Kore
MK092481.1_PPV7/Chin
MK092479.1_PPV7/Chin
MG543470.1_PPV7/Chin
MG543467.1_PPV7/Chin
MG696111.1_PPV7/Chin
MG696112.1_PPV7/Chin
MG914435.1_PPV7/Swed
MH422963.1_PPV7/Kore
MH422964.1_PPV7/Kore
MH422966.1_PPV7/Kore
MH422967.1_PPV7/Kore
MH817776.1_PPV7/Kore
MH817777.1_PPV7Korea
MK092477.1_PPV7/Chin
MK092480.1_PPV7/Chin
MK092482.1_PPV7/Chin
MK092483.1_PPV7/Chin
MK092484.1_PPV7/Chin
MK092485.1_PPV7/Chin
MK092486.1_PPV7/Chin
MK092487.1_PPV7/Chin
MK092488.1_PPV7/Chin
MK092489.1_PPV7/Chin
MK092490.1_PPV7/Chin
MK092491.1_PPV7/Chin
MK092492.1_PPV7/Chin
MK092493.1_PPV7/Chin
MK092495.1_PPV7/Chin
MK092496.1_PPV7/Chin
MK484100.1_PPV7/Chin
MK484101.1_PPV7/Chin
MK484102.1_PPV7/Chin
MN515032.1_PPV7/Braz
MT747168_PPV7/COL/Cu
MT758696_PPV7/Col/An
MT758697_PPV7/Col/Co
MT758695_PPV7/Col/Ri
730
740
750
760
770
780
790
80
KU563733.1_PPV7/USA-
KY996756.1_PPV7/Chin
KY996757.1_PPV7/Chin
KY996758.1_PPV7/Chin
MG543456.1_PPV7/Chin
MG543457.1_PPV7/Chin
MG543458.1_PPV7/Chin
MG543459.1_PPV7/Chin
MG543460.1_PPV7/Chin
MG543461.1_PPV7/Chin
MG543462.1_PPV7/Chin
MG543463.1_PPV7/chin
MG543464.1_PPV7/Chin
MG543465.1_PPV7/Chin
MG543466.1_PPV7/Chin
MG543469.1_PPV7/Chin
MG543471.1_PPV7/Chin
MG902949.1_PPV7/Chin
MG543472.1_PPV7/Chin
MH422965.1_PPV7/Kore
MH422962.1_PPV7/Kore
MK092481.1_PPV7/Chin
MK092479.1_PPV7/Chin
MG543470.1_PPV7/Chin
MG543467.1_PPV7/Chin
MG696111.1_PPV7/Chin
MG696112.1_PPV7/Chin
MG914435.1_PPV7/Swed
MH422963.1_PPV7/Kore
MH422964.1_PPV7/Kore
MH422966.1_PPV7/Kore

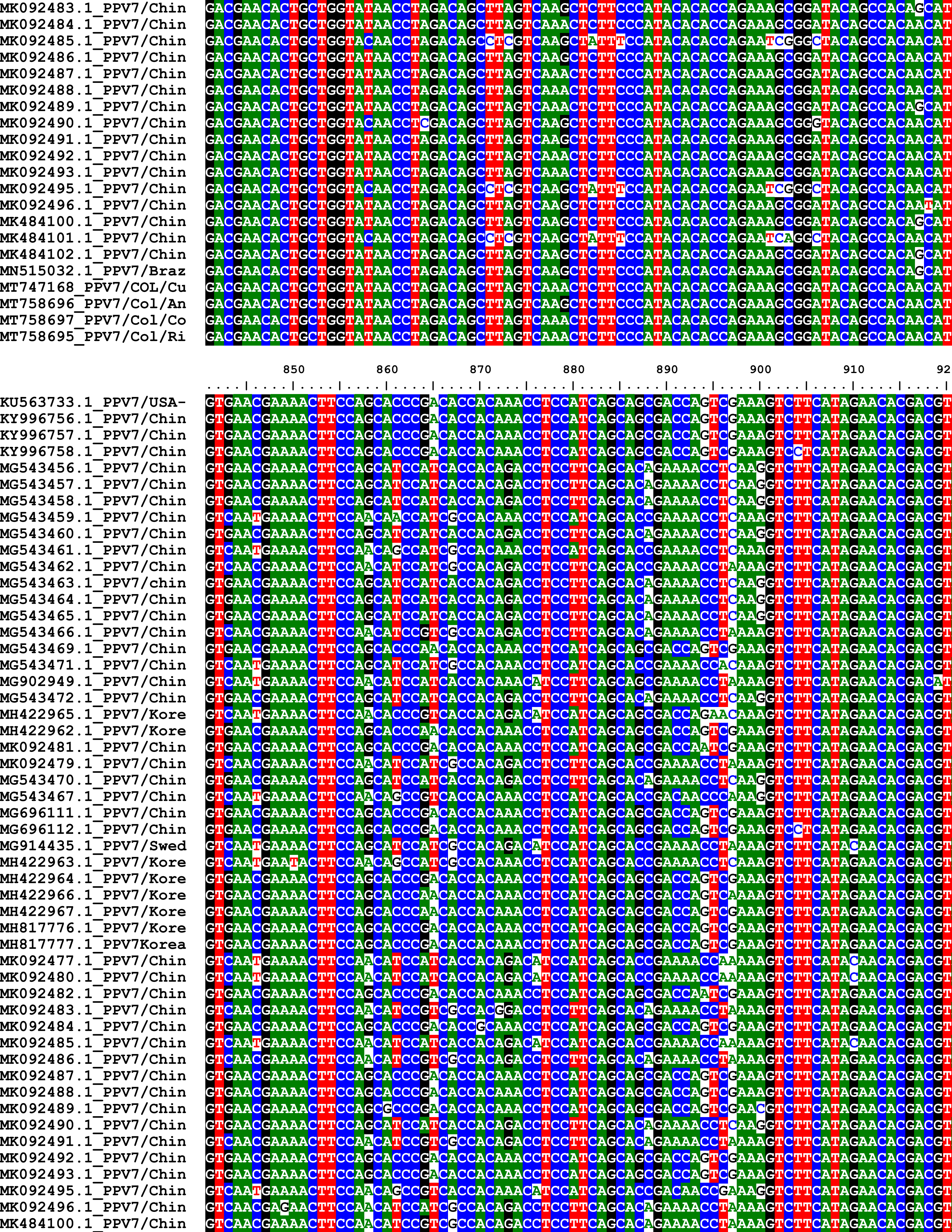
MK092483.1_PPV7/Chin
MK092484.1_PPV7/Chin
MK092485.1_PPV7/Chin
MK092486.1_PPV7/Chin
MK092487.1_PPV7/Chin
MK092488.1_PPV7/Chin
MK092489.1_PPV7/Chin
MK092490.1_PPV7/Chin
MK092491.1_PPV7/Chin
MK092492.1_PPV7/Chin
MK092493.1_PPV7/Chin
MK092495.1_PPV7/Chin
MK092496.1_PPV7/Chin
MK484100.1_PPV7/Chin
MK484101.1_PPV7/Chin
MK484102.1_PPV7/Chin
MN515032.1_PPV7/Braz
MT747168_PPV7/COL/Cu
MT758696_PPV7/Col/An
MT758697_PPV7/Col/Co
MT758695_PPV7/Col/Ri
850
860
870
880
890
900
910
92
KU563733.1_PPV7/USA-
KY996756.1_PPV7/Chin
KY996757.1_PPV7/Chin
KY996758.1_PPV7/Chin
MG543456.1_PPV7/Chin
MG543457.1_PPV7/Chin
MG543458.1_PPV7/Chin
MG543459.1_PPV7/Chin
MG543460.1_PPV7/Chin
MG543461.1_PPV7/Chin
MG543462.1_PPV7/Chin
MG543463.1_PPV7/chin
MG543464.1_PPV7/Chin
MG543465.1_PPV7/Chin
MG543466.1_PPV7/Chin
MG543469.1_PPV7/Chin
MG543471.1_PPV7/Chin
MG902949.1_PPV7/Chin
MG543472.1_PPV7/Chin
MH422965.1_PPV7/Kore
MH422962.1_PPV7/Kore
MK092481.1_PPV7/Chin
MK092479.1_PPV7/Chin
MG543470.1_PPV7/Chin
MG543467.1_PPV7/Chin
MG696111.1_PPV7/Chin
MG696112.1_PPV7/Chin
MG914435.1_PPV7/Swed
MH422963.1_PPV7/Kore
MH422964.1_PPV7/Kore
MH422966.1_PPV7/Kore
MH422967.1_PPV7/Kore
MH817776.1_PPV7/Kore
MH817777.1_PPV7Korea
MK092477.1_PPV7/Chin
MK092480.1_PPV7/Chin
MK092482.1_PPV7/Chin
MK092483.1_PPV7/Chin
MK092484.1_PPV7/Chin
MK092485.1_PPV7/Chin
MK092486.1_PPV7/Chin
MK092487.1_PPV7/Chin
MK092488.1_PPV7/Chin
MK092489.1_PPV7/Chin
MK092490.1_PPV7/Chin
MK092491.1_PPV7/Chin
MK092492.1_PPV7/Chin
MK092493.1_PPV7/Chin
MK092495.1_PPV7/Chin
MK092496.1_PPV7/Chin
MK484100.1_PPV7/Chin

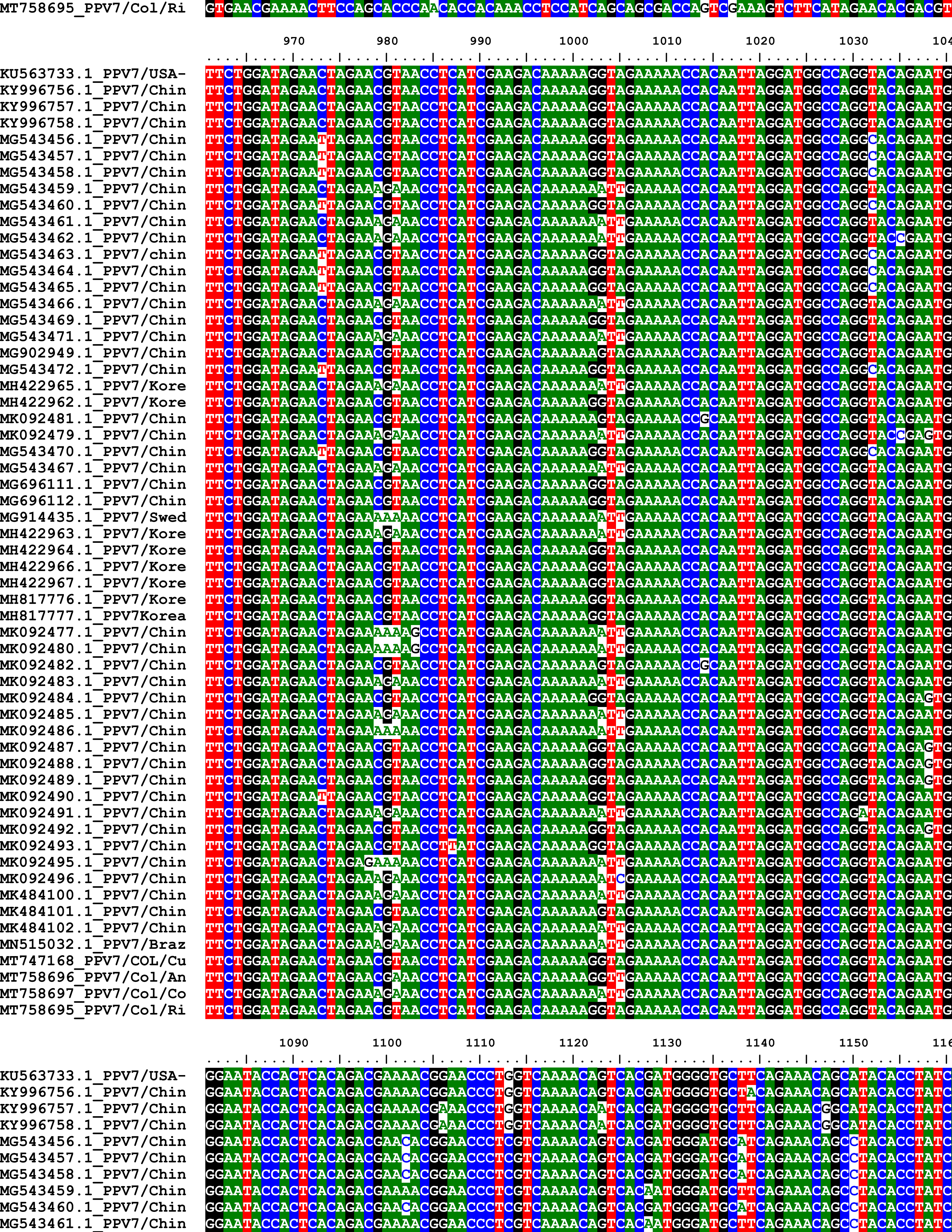
MT758695_PPV7/Col/Ri
970 980 990 1000 1010 1020 1030
KU563733.1_PPV7/USA-
KY996756.1_PPV7/Chin
KY996757.1_PPV7/Chin
KY996758.1_PPV7/Chin
MG543456.1_PPV7/Chin
MG543457.1_PPV7/Chin
MG543458.1_PPV7/Chin
MG543459.1_PPV7/Chin
MG543460.1_PPV7/Chin
MG543461.1_PPV7/Chin
MG543462.1_PPV7/Chin
MG543463.1_PPV7/chin
MG543464.1_PPV7/Chin
MG543465.1_PPV7/Chin
MG543466.1_PPV7/Chin
MG543469.1_PPV7/Chin
MG543471.1_PPV7/Chin
MG902949.1_PPV7/Chin
MG543472.1_PPV7/Chin
MH422965.1_PPV7/Kore
MH422962.1_PPV7/Kore
MK092481.1_PPV7/Chin
MK092479.1_PPV7/Chin
MG543470.1_PPV7/Chin
MG543467.1_PPV7/Chin
MG696111.1_PPV7/Chin
MG696112.1_PPV7/Chin
MG914435.1_PPV7/Swed
MH422963.1_PPV7/Kore
MH422964.1_PPV7/Kore
MH422966.1_PPV7/Kore
MH422967.1_PPV7/Kore
MH817776.1_PPV7/Kore
MH817777.1_PPV7Korea
MK092477.1_PPV7/Chin
MK092480.1_PPV7/Chin
MK092482.1_PPV7/Chin
MK092483.1_PPV7/Chin
MK092484.1_PPV7/Chin
MK092485.1_PPV7/Chin
MK092486.1_PPV7/Chin
MK092487.1_PPV7/Chin
MK092488.1_PPV7/Chin
MK092489.1_PPV7/Chin
MK092490.1_PPV7/Chin
MK092491.1_PPV7/Chin
MK092492.1_PPV7/Chin
MK092493.1_PPV7/Chin
MK092495.1_PPV7/Chin
MK092496.1_PPV7/Chin
MK484100.1_PPV7/Chin
MK484101.1_PPV7/Chin
MK484102.1_PPV7/Chin
MN515032.1_PPV7/Braz
MT747168_PPV7/COL/Cu
MT758696_PPV7/Col/An
MT758697_PPV7/Col/Co
MT758695_PPV7/Col/Ri
1090 1100 1110 1120 1130 1140 1150
KU563733.1_PPV7/USA-
KY996756.1_PPV7/Chin
KY996757.1_PPV7/Chin
KY996758.1_PPV7/Chin
MG543456.1_PPV7/Chin
MG543457.1_PPV7/Chin
MG543458.1_PPV7/Chin
MG543459.1_PPV7/Chin
MG543460.1_PPV7/Chin
MG543461.1_PPV7/Chin

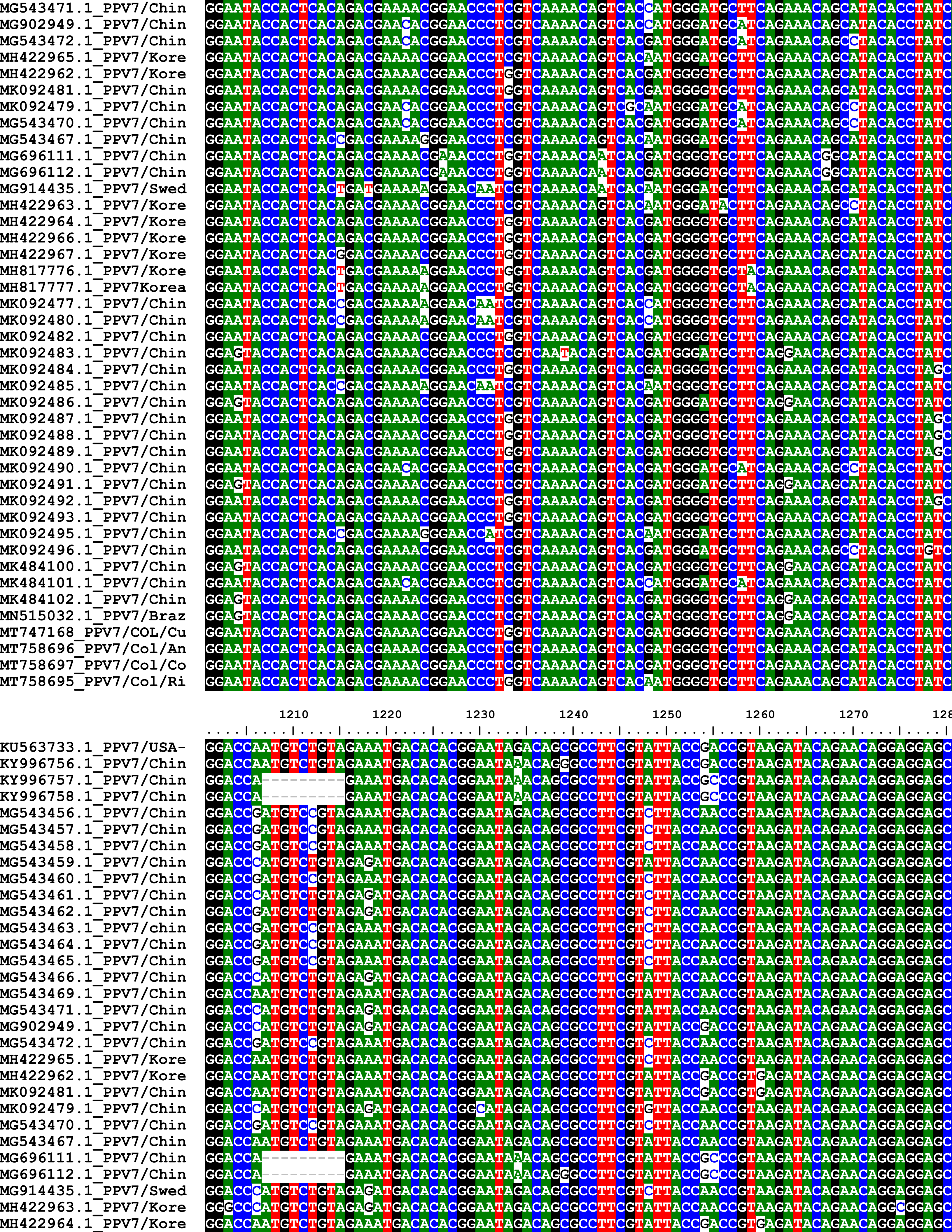

MG543471.1_PPV7/Chin
MG902949.1_PPV7/Chin
MG543472.1_PPV7/Chin
MH422965.1_PPV7/Kore
MH422962.1_PPV7/Kore
MK092481.1_PPV7/Chin
MK092479.1_PPV7/Chin
MG543470.1_PPV7/Chin
MG543467.1_PPV7/Chin
MG696111.1_PPV7/Chin
MG696112.1_PPV7/Chin
MG914435.1_PPV7/Swed
MH422963.1_PPV7/Kore
MH422964.1_PPV7/Kore
MH422966.1_PPV7/Kore
MH422967.1_PPV7/Kore
MH817776.1_PPV7/Kore
MH817777.1_PPV7Korea
MK092477.1_PPV7/Chin
MK092480.1_PPV7/Chin
MK092482.1_PPV7/Chin
MK092483.1_PPV7/Chin
MK092484.1_PPV7/Chin
MK092485.1_PPV7/Chin
MK092486.1_PPV7/Chin
MK092487.1_PPV7/Chin
MK092488.1_PPV7/Chin
MK092489.1_PPV7/Chin
MK092490.1_PPV7/Chin
MK092491.1_PPV7/Chin
MK092492.1_PPV7/Chin
MK092493.1_PPV7/Chin
MK092495.1_PPV7/Chin
MK092496.1_PPV7/Chin
MK484100.1_PPV7/Chin
MK484101.1_PPV7/Chin
MK484102.1_PPV7/Chin
MN515032.1_PPV7/Braz
MT747168_PPV7/COL/Cu
MT758696_PPV7/Col/An
MT758697_PPV7/Col/Co
MT758695_PPV7/Col/Ri
1210
1220
1230
1240
1250
1260
1270
KU563733.1_PPV7/USA-
KY996756.1_PPV7/Chin
KY996757.1_PPV7/Chin
KY996758.1_PPV7/Chin
MG543456.1_PPV7/Chin
MG543457.1_PPV7/Chin
MG543458.1_PPV7/Chin
MG543459.1_PPV7/Chin
MG543460.1_PPV7/Chin
MG543461.1_PPV7/Chin
MG543462.1_PPV7/Chin
MG543463.1_PPV7/chin
MG543464.1_PPV7/Chin
MG543465.1_PPV7/Chin
MG543466.1_PPV7/Chin
MG543469.1_PPV7/Chin
MG543471.1_PPV7/Chin
MG902949.1_PPV7/Chin
MG543472.1_PPV7/Chin
MH422965.1_PPV7/Kore
MH422962.1_PPV7/Kore
MK092481.1_PPV7/Chin
MK092479.1_PPV7/Chin
MG543470.1_PPV7/Chin
MG543467.1_PPV7/Chin
MG696111.1_PPV7/Chin
MG696112.1_PPV7/Chin
MG914435.1_PPV7/Swed
MH422963.1_PPV7/Kore
MH422964.1_PPV7/Kore

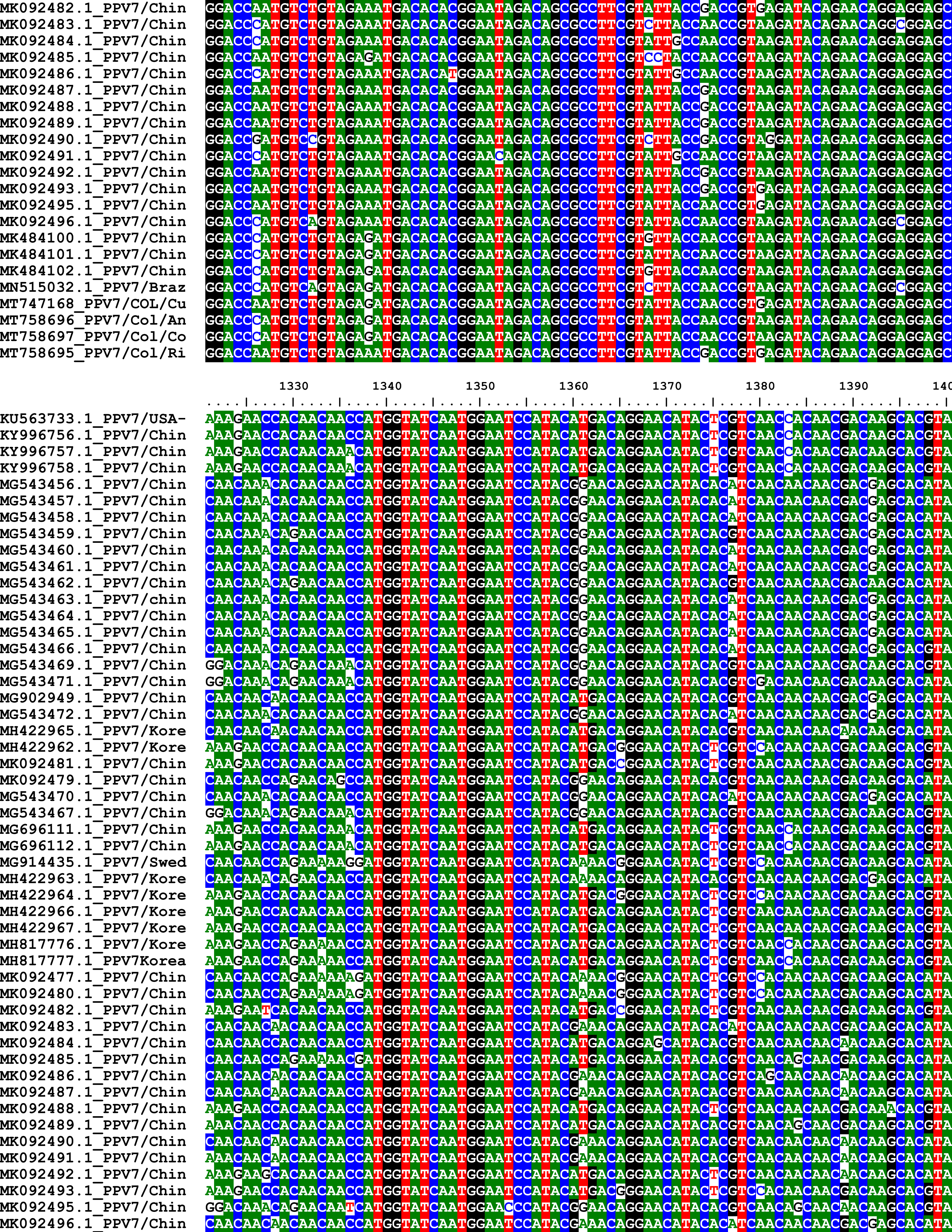

MK092482.1_PPV7/Chin
MK092483.1_PPV7/Chin
MK092484.1_PPV7/Chin
MK092485.1_PPV7/Chin
MK092486.1_PPV7/Chin
MK092487.1_PPV7/Chin
MK092488.1_PPV7/Chin
MK092489.1_PPV7/Chin
MK092490.1_PPV7/Chin
MK092491.1_PPV7/Chin
MK092492.1_PPV7/Chin
MK092493.1_PPV7/Chin
MK092495.1_PPV7/Chin
MK092496.1_PPV7/Chin
MK484100.1_PPV7/Chin
MK484101.1_PPV7/Chin
MK484102.1_PPV7/Chin
MN515032.1_PPV7/Braz
MT747168_PPV7/COL/Cu
MT758696_PPV7/Col/An
MT758697_PPV7/Col/Co
MT758695_PPV7/Col/Ri
1330
1340
1350
1360
1370
1380
1390
KU563733.1_PPV7/USA-
KY996756.1_PPV7/Chin
KY996757.1_PPV7/Chin
KY996758.1_PPV7/Chin
MG543456.1_PPV7/Chin
MG543457.1_PPV7/Chin
MG543458.1_PPV7/Chin
MG543459.1_PPV7/Chin
MG543460.1_PPV7/Chin
MG543461.1_PPV7/Chin
MG543462.1_PPV7/Chin
MG543463.1_PPV7/chin
MG543464.1_PPV7/Chin
MG543465.1_PPV7/Chin
MG543466.1_PPV7/Chin
MG543469.1_PPV7/Chin
MG543471.1_PPV7/Chin
MG902949.1_PPV7/Chin
MG543472.1_PPV7/Chin
MH422965.1_PPV7/Kore
MH422962.1_PPV7/Kore
MK092481.1_PPV7/Chin
MK092479.1_PPV7/Chin
MG543470.1_PPV7/Chin
MG543467.1_PPV7/Chin
MG696111.1_PPV7/Chin
MG696112.1_PPV7/Chin
MG914435.1_PPV7/Swed
MH422963.1_PPV7/Kore
MH422964.1_PPV7/Kore
MH422966.1_PPV7/Kore
MH422967.1_PPV7/Kore
MH817776.1_PPV7/Kore
MH817777.1_PPV7Korea
MK092477.1_PPV7/Chin
MK092480.1_PPV7/Chin
MK092482.1_PPV7/Chin
MK092483.1_PPV7/Chin
MK092484.1_PPV7/Chin
MK092485.1_PPV7/Chin
MK092486.1_PPV7/Chin
MK092487.1_PPV7/Chin
MK092488.1_PPV7/Chin
MK092489.1_PPV7/Chin
MK092490.1_PPV7/Chin
MK092491.1_PPV7/Chin
MK092492.1_PPV7/Chin
MK092493.1_PPV7/Chin
MK092495.1_PPV7/Chin
MK092496.1_PPV7/Chin

```
MT758697_PPV7/Col/Co   CAACAACCACAACAACCATGGTATCAATGGAATCCATACGGAACAGGAACATACACATCAACAACAACGACAAGCACATA
MT758695_PPV7/Col/Ri   AAAGAACCACAACAACCATGGTATCAATGGAATCCATACATGACGGGAACATACTCGTCAACAACAACGACAAGCACGTA
```
