# Supplementary material for: The first report of porcine parvovirus 7 (PPV7) in Colombia demonstrates the presence of variants associated with modifications at the level of the VP2-capsid protein
Source: PLoS One. 2021 Dec 16;16(12):e0258311. doi: 10.1371/journal.pone.0258311 (PMC8675767; doi:10.1371/journal.pone.0258311)
Supplement: S1 Table — (DOCX) [file pone.0258311.s006.docx]

**S1 Table. Summary of PPV7 reference sequence used in this study**

| Number | Name | Country | GenBank accession number |
| --- | --- | --- | --- |
| 1 | AHhf | China | MK484102 |
| 2 | AHbz | China | MK484100 |
| 3 | AHmas | China | MK484101 |
| 4 | GX2 | China | MG543456 |
| 5 | GX3 | China | MG543457 |
| 6 | GX5 | China | MG543458 |
| 7 | GX6 | China | MG543459 |
| 8 | GX28 | China | MG543460 |
| 9 | GX29 | China | MG543461 |
| 10 | GX30 | China | MG543462 |
| 11 | GX31 | China | MG543463 |
| 12 | GX32 | China | MG543464 |
| 13 | GX34 | China | MG543465 |
| 14 | GX35 | China | MG543466 |
| 15 | GX44 | China | MG543467 |
| 16 | GX48 | China | MG543469 |
| 17 | GX47 | China | MG543470 |
| 18 | GX49 | China | MG543471 |
| 19 | GX50 | China | MG543472 |
| 20 | Strain37 | China | MG902949 |
| 21 | GD-2014-1 | China | KY996756 |
| 22 | GD-2014-2 | China | KY996757 |
| 23 | GD-2014-3 | China | KY996758 |
| 24 | FJFZ2017 | China | MG696111 |
| 25 | FJLY2017 | China | MG696112 |
| 26 | 87 CLON2 | China | MK092482 |
| 27 | JX10 | China | MK092493 |
| 28 | DJH11 | China | MK092484 |
| 29 | DJH12 | China | MK092485 |
| 30 | DJH13 | China | MK092486 |
| 31 | DJH14 | China | MK092487 |
| 32 | DJH19 | China | MK092488 |
| 33 | DJH20 | China | MK092489 |
| 34 | DJH23 | China | MK092490 |
| 35 | DJH24 | China | MK092491 |
| 36 | DJH26 | China | MK092492 |
| 37 | 87 CLON1 | China | MK092481 |
| 38 | PPV7 –LB4 | China | MK092483 |
| 39 | PPV7 -77 | China | MK092479 |
| 40 | JX21 | China | MK092495 |
| 41 | PPV7 -55 | China | MK092477 |
| 42 | PPV7 -80 | China | MK092480 |
| 43 | JX38 | China | MK092496 |
| 44 | N141 | Korea | MH717776 |
| 45 | N133 | Korea | MH717777 |
| 46 | KF1 | Korea | MH422962 |
| 47 | KF2 | Korea | MH422963 |
| 48 | KF3 | Korea | MH422964 |
| 49 | KF4 | Korea | MH422965 |
| 50 | KF5 | Korea | MH422966 |
| 51 | KF6 | Korea | MH422967 |
| 52 | SWE20 | Sweden | MG914435 |
| 53 | 42 | USA | KU563733 |
| 54 | BR_RSPPV7 | Brazil | MN515032 |
